# Environmental DNA reveals diversity and abundance of *Alternaria* species in neighbouring heterogeneous landscapes in Worcester, UK

**DOI:** 10.1007/s10453-022-09760-9

**Published:** 2022-10-23

**Authors:** Godfrey Philliam Apangu, Carl Alexander Frisk, Geoffrey M. Petch, Lucia Muggia, Alberto Pallavicini, Mary Hanson, Carsten Ambelas Skjøth

**Affiliations:** 1 School of Science and the Environment, University of Worcester, Henwick Grove, WR2 6AJ Worcester UK; 2 Department of Life Sciences, University of Trieste, Via Giorgieri 10, 34127 Trieste, Italy; 3 Present Address: Protecting Crops and the Environment, Rothamsted Research, West Common, Harpenden, AL5 2JQ Hertfordshire UK; 4 Present Address: Department of Urban Greening and Vegetation Ecology, Norwegian Institute of Bioeconomy Research, Ås, Norway; 5 Department of Environmental Science, Aarhus University, Frederiksborgvej 399, 4000 Roskilde, Denmark

**Keywords:** Metabarcoding, Microscopy, Fungi, ITS1, ITS2, Unmanaged grassland

## Abstract

*Alternaria* is a pathogenic and allergenic fungus affecting 400 plant species and 334 million people globally. This study aimed at assessing the diversity of *Alternaria* species in airborne samples collected from closely located (7 km apart) and heterogeneous sites (rural, urban and unmanaged grassland) in Worcester and Lakeside, the UK. A secondary objective was to examine how the ITS1 subregion varies from ITS2 in *Alternaria* species diversity and composition. Airborne spores were collected using Burkard 7-day and multi-vial Cyclone samplers for the period 5 July 2016–9 October 2019. Air samples from the Cyclone were amplified using the ITS1and ITS2 subregions and sequenced using Illumina MiSeq platform whereas those from the Burkard sampler were identified and quantified using optical microscopy. Optical microscopy and eDNA revealed a high abundance of *Alternaria* in the rural, urban and unmanaged sites. ITS1 and ITS2 detected five and seven different *Alternaria* species at the three sampling sites, respectively. *A. dactylidicola*, *A. metachromatica* and *A. infectoria* were the most abundant. The rural, urban and unmanaged grassland sites had similar diversity (PERMANOVA) of the species due to similarity in land use and proximity of the sites. Overall, the study showed that heterogeneous and neighbouring sites with similar land uses can have similar *Alternaria* species. It also demonstrated that an eDNA approach can complement the classical optical microscopy method in providing more precise information on fungal species diversity in an environment for targeted management. Similar studies can be replicated for other allergenic and pathogenic fungi.

**Supplementary Information:**

The online version contains supplementary material available at 10.1007/s10453-022-09760-9.

## Introduction

*Alternaria* is a saprophytic, endophytic and pathogenic fungus with many species ubiquitous in nature (Rotem, 1994). Precisely 275 *Alternaria* species, having specific or wide host range, are pathogenic to nearly 400 plant species including those in the Poaceae, Cucurbitaceae, Brassicaceae and Solanaceae families (Lee et al., 2015; Meena et al., 2017b; Seifert & Gams, 2011; Simmons, 2007; Skjøth et al., 2016). Crop yield losses of up to 80% have been attributed to *Alternaria* diseases in several years of production (Maude & Humpherson-Jones, 1980; Nowicki et al., 2012). *Alternaria* is also an allergenic fungus affecting up to 70% of mould-allergic patients (Sanchez & Bush, 2001). About 37 *Alternaria* species are known to cause allergy and other respiratory tract disorders such as alveolitis, rhinitis, bronchitis and eczema (Hong et al., 2005; Meena et al., 2017a; Seifert & Gams, 2011; Skjøth et al., 2016). Inhalation of allergenic *Alternaria* spores triggers severe and potentially fatal asthma in sensitised individuals, most especially children (Gabriel et al., 2015; Sanchez & Bush, 2001). Often, the forecast advice to allergy sufferers and asthmatics for prevention is based on the daily *Alternaria* spp. spore concentration in the air in general (Grinn-Gofroń et al., 2019). This approach does not provide information on the particular species that are most abundant in the air thus leading to allergic individuals and asthmatics taking measures including medications based on basic forecast information.

Understanding fungal species diversity, e.g. in *Alternaria* spp. in an area helps to provide more targeted information on spore forecast and for targeted management of fungal diseases. However, aerobiological studies mostly use the classical microscopic method that relies on morphological features of the most abundant and recognisable fungal spores to provide spore data at the genus level (Banchi et al., 2018; Pashley et al., 2012; Sharma et al., 2015). The development of DNA metabarcoding alongside high-throughput sequencing (HTS) technology has enabled an increased number of studies in airborne plant diversity (Hebert et al., 2003; Joly & Peuch, 2012). However, few of such studies exist on airborne fungal diversity (Banchi et al., 2018). Moreover, some of the metabarcoding fungal diversity studies focus mostly on the genera level leaving out species diversity (Banchi et al., 2020a, 2020b; Rosa et al., 2020a, b Fort et al., 2016; Tordoni et al., 2021) which could otherwise provide cues on specific fungal species relevant for the prevention of allergy and pathological diseases.

In this study, we aimed to assess the diversity, richness, composition and abundance of *Alternaria* species in airborne samples collected from rural, urban and unmanaged sites that are located approximately 7 km apart in Worcester and Lakeside Container/Lakeside Circle, the UK. This was addressed by examining the hypothesis that there is a low diversity and high abundance of *Alternaria* species in nearby sites with similar land uses. Airborne eDNA and HTS fungal diversity studies focussing on both urban and rural sites are very limited (Bowers et al., 2013; Leppänen et al., 2018; Lin et al., 2018; Wady et al., 2004) and yet cases of allergy and asthma are high in such sites (Mitakakis et al., 2001; von Mutius, 2008). Moreover, there are no such eDNA and HTS studies of fungal diversity in unmanaged sites, e.g. grassland. Therefore, we sequenced the eDNA from the air samples using both the internal transcribed spacer (ITS1 and ITS2) subregions and the Illumina MiSeq platform.

## Materials and methods

### Spore sampling

**Fig. 1:**
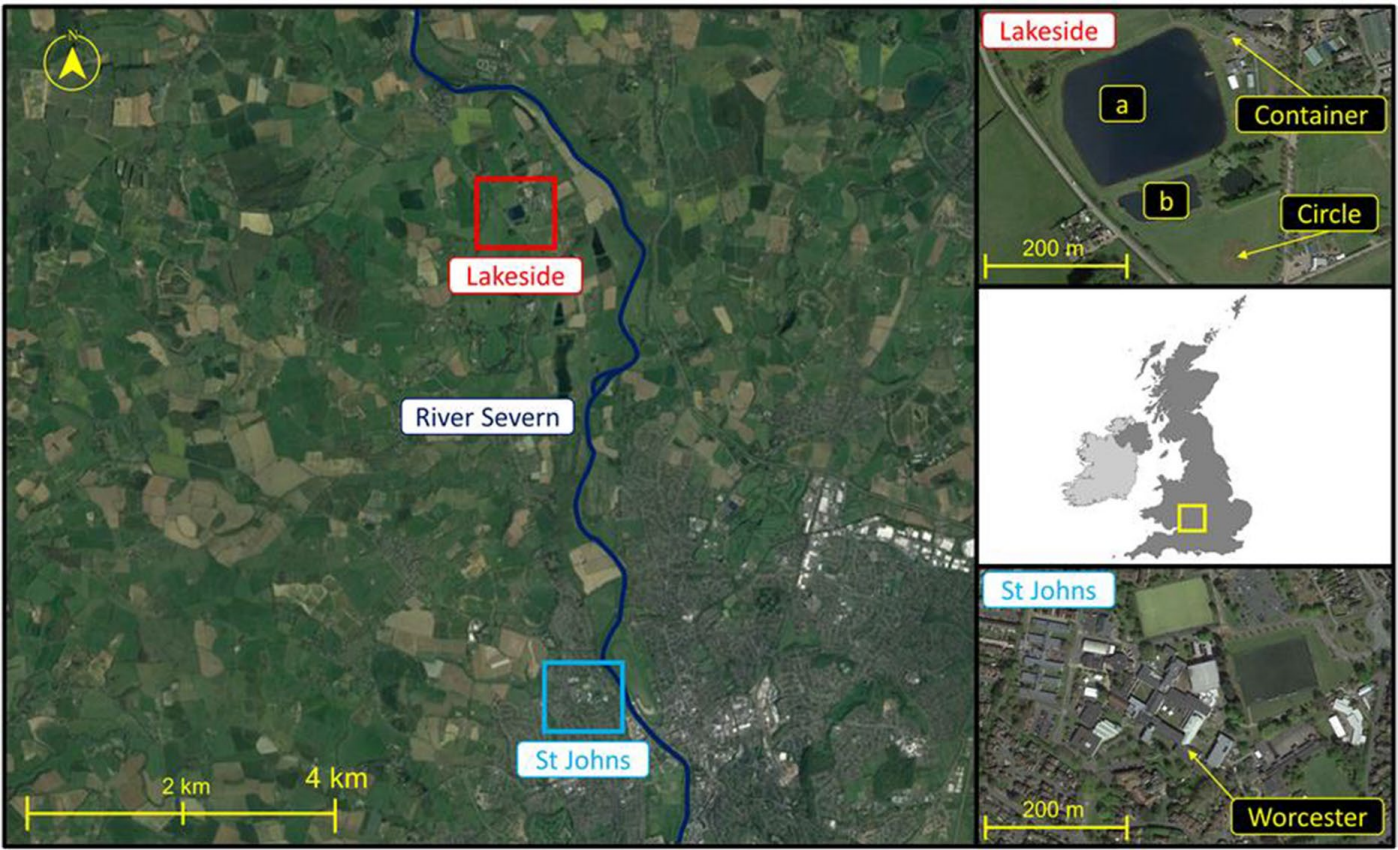
Spore sampling and meteorological station sites of Lakeside Container, Lakeside Circle and Worcester. Also indicated on the map are the (**a**) Lake near Lakeside Container and (**b**) Lake near Lakeside Circle and River Severn (dark blue spiralling feature on left map). Lakeside and St Johns maps on the right are enlargements of their corresponding map sections on the left

*Alternaria* spores were sampled using a Hirst-type Burkard 7-day volumetric spore trap (Hirst, 1952) and a Burkard automatic multi-vial Cyclone sampler (Burkard Manufacturing, UK). Samples were collected at St John’s campus (hereafter Worcester) and Lakeside campus (hereafter Lakeside Container and Lakeside Circle) of the University of Worcester (Fig. 1). Air samples were collected during the main *Alternaria* season at Worcester and Lakeside Container from 5 July 2016 to 9 October 2019 (Table S1). Sampling heights were set following the recommendations on the height of pollen/spore sampling (Galán et al., 2014). The Burkard 7-day and Cyclone samplers at Worcester were placed 10 m above ground level (AGL) on the rooftop of the Edward Elgar (EE) building (52.1970 N, − 2.2421 E, e.g. Sadyś et al., 2014) to capture spores at a regional scale. The Burkard 7-day and Cyclone samplers collected air at a rate of 10 L/min and 16.5 L/min, respectively (Hirst, 1952) and the flow rate of the samplers was checked weekly. The airborne particles were deposited by impaction on a tape coated with a thin film of petroleum jelly/wax mixture. The samplers at Worcester were unloaded weekly at 9:00 am while those at Lakeside Container and Lakeside Circle were emptied at 14:00 pm and their spore data every 2-h was later matched with those at Worcester. Worcester is an urban area surrounded by agricultural areas comprising permanent orchards for fruit and cider production (Sadyś et al., 2014), crops under rotation (Sadyś et al., 2015), grasslands and pasture within the public parks (Sadyś et al., 2016) and small woodlands (Skjøth et al., 2015). The nearest crop fields to the trap in Worcester were half a km away in a westerly direction (Apangu et al., 2020).

The Burkard and Cyclone samplers at Lakeside Container (52.2544 N, − 2.2537 E) were placed on a container at 4 m AGL. The Lakeside Container sampling site was in a rural environment and comprised mixed arable crop fields, permanent pastures, animal paddocks and patches of trees. The immediate vicinity of the sampler had no source of *Alternaria* spores and it was surrounded by buildings in the south, non-vegetated areas such as hard standing, roadways, and a man-made lake in the west, pine trees in the north and other trees and large areas of amenity grassland in the east. The area outside the vicinity of the sampler was comprised of crop fields under rotation and woodlands.

The third set of Burkard and Cyclone samplers collected spores at Lakeside Circle (52.2516 N, − 2.2535 E; Fig. 1) from 12 July 2018 to 9 October 2019 (Table S1). Historically, the area was an arable farm with annual rotations until after the 2016 crop harvest when it became amenity grassland. A circle, centrally on the field and with a diameter of 50 m was created in the grassland in 2018. The major grass and herb species growing in the circular grassland included *Festuca rubra*, *Lolium perenne* and *Trifolium repens*. Grasses such as *Festuca* are known hosts of *Alternaria* spp. (Awad, 2005; Wilson et al., 2014). Nitrogen, phosphorus, potassium (NPK) fertiliser mixture was applied to the grassland in the circle at the start of the observation period in spring 2018 to support root establishment and vigorous growth of the grasses (Leuschner et al., 2013). Thereafter, the grass circle remained unmanaged to enable natural production and release of fungal spores including *Alternaria*. A pair of Burkard 7-day and Cyclone samplers were placed 2.5 m AGL on a mast located in the centre of the circle. The grassland area outside the circle was mowed regularly (~ every 3 weeks) to ensure a green and well-growing grass patch without large amounts of decomposing plant material outside the circle. Lakeside Container and Lakeside Circle were approximately 310 m apart and both were approximately 7 km away from Worcester. The landscape outside the established grassland comprised mixed arable farms of rotational crop fields, permanent pastures and animal paddocks.

### Microscopic *Alternaria* spp. spore identification

During slide preparation, the tape was cut into 7 pieces (each piece 48 mm long), each corresponding to one of the seven days in a week, and mounted on a microscopic slide (Sadyś et al., 2014). Slides were prepared according to a standard procedure used for more than 50 years in England and other European countries (Adams-Groom et al., 2002; BAF, 1995; Kasprzyk, 2008; Skjøth et al., 2008, 2015). *Alternaria* spores were identified to genus level and counted using the 12 transverse method at 400× magnification, an approach used for fungal spore monitoring in Worcester, UK (Apangu et al., 2020), Denmark (Skjøth et al., 2012) and Hungary (Paldy et al., 2014). The daily (24 h) mean *Alternaria* spore concentrations were expressed as spores/m^3^ of air by multiplying the microscopic spore counts with previously calculated correction factors (Lacey & Allit, 1995).

### Cultivation of *A. alternata* conidia

*A*. *alternata* spores were cultivated in the Lab to be used in a mock community. Conidia of *A. alternata* procured from Fisher Scientific UK were cultured on potato dextrose agar for 23 days at 23 °C, similar to Smith et al. (2012). To harvest spores, sterile water (10 mL) was added to the culture petri dish and spore suspensions were obtained by gently scraping the surface of the culture using a sterile L-shaped spreader. Five mL of the spore suspension was drawn into a clean and sterile 50 mL centrifuge tube. The mycelial extract was recovered after 5 min of centrifugation at 2500 rpm. The supernatant was discarded and the pellet was transferred into a clean 2 mL microcentrifuge tube and resuspended in 1 mL sterile water.

### DNA extraction from air samples and culture material

The daily air samples were pooled every seven consecutive days of sampling according to a pre-planned sampling date arrangement (Table S1), similar to Brennan et al. (2019). DNA was isolated from the air samples and the *A. alternata* culture material using a commercial protocol (Fast DNA spin kit for soil; MP Biomedicals), similar to previous studies (Chen et al., 2020; Degois et al., 2019; Ettenauer et al., 2012; Fröhlich-Nowoisky et al., 2012). The sample for 28/07/2018 at Lakeside circle, which comprised water, typically caused by heavy rain episodes or fog, was excluded from extraction. The concentration of DNA in the samples was quantified using a Nanodrop 2000c spectrophotometer instrument (Fisher Scientific, UK), similar to previous studies (Degois et al., 2019; Ettenauer et al., 2012; Shokere et al., 2009). The DNA was stored at − 20 °C for subsequent analyses.

### Mock community

A large collection of pollen and spores of different plant and fungal species were originally acquired as clean reference samples from commercial companies and stored in a refrigerator at 4 °C in the Charles Darwin laboratory of the University of Worcester. Aiming at containing both allergenic pollen and allergenic and pathogenic spores, small amounts from the clean reference samples were pooled to form a mock community. The mock community sample consisted of *Cladosporium* sp., *Alternaria* sp., *Dactylis glomerata, Lolium perenne, Artemisia vulgaris*, *Quercus ilex, Quercus robur, Alnus glutinosa, Betula pendula, Corylus avenella, Phleum pratense, Urtica dioica, Platanus × hispanica* and *A. alternata,* similar to previous metabarcoding studies on fungal spores (Aguayo et al., 2018; Heeger et al., 2018; Pauvert et al., 2019)*.* The species such as *L. perenne, U. dioica* and *Cladosporium* sp. were included because of their overlapping seasonality with *Alternaria* while *A. glutinosa and C. avenella* were added due to their seasonal differences with *Alternaria.* DNA was extracted from the mock community sample using the Fast DNA spit kit protocol, as for the air samples above. The mock community DNA was then stored at − 20 °C for downstream analyses.

### Preparation of air samples and mock community for metabarcoding

For each site, the 7-day pooled samples were re-pooled for each sampling season to form a composite sample (Table S2). Ten re-pooled air samples and the mock community were used in Illumina sequencing. To prevent any contamination, microcentrifuge tubes were autoclaved before re-pooling the DNA of the air samples. Four µL of DNA were drawn from each weekly pooled air sample per season and added to the sterile microcentrifuge tube to form a composite sample. Meanwhile, 50 µL of the total DNA of the mock community was used for sequencing. The Nanodrop 2000c spectrophotometer was used for measuring the concentration of DNA in each sample. DNA of each sample and mock community was suspended in TE buffer [a mixture of 1 mM Ethylenediaminetetraacetic acid (EDTA) and 10 mM Tris(Hydroxymethyl)] to prevent DNA degradation and stored at 4 °C before shipment to Eurofins Genomics for ITS1/ITS2 amplification and sequencing, as recommended by Clasen et al. (2020). DES (DNase/Pyrogen-free water; 50 µL) was used as a negative control in the sequencing process.

### PCR amplification and amplicon sequencing

Eurofins Genomics, Europe Sequencing GmbH, Konstanz, Germany synthesised the primers, performed the PCR assays and amplicon sequencing of the air samples and the mock community, similar to previous studies (Naveed et al., 2016; Nicolaisen et al., 2017; Rossmann et al., 2021; Senés-Guerrero & Schüßler, 2016). The PCR assay conditions were similar to Nicolaisen et al. (2017). The ITS is one of the most widely sequenced DNA regions in fungi and is the universally accepted genetic barcode for fungi (Abrego et al., 2018; Schoch et al., 2012). Therefore, we amplified 300 bp each of ITS1 and ITS2 regions using the universal primer pairs fwd-5′-GGAAGTAAAAGTCGTAACAAGG-3′; rev-5′-GCTGCGTTCTTCATCGATGC-3′ and fwd-5′-GCATCGATGAAGAACGCAGC-3′; rev-5′-TCCTCCGCTTATTGATATGC-3′, respectively, targeting specifically fungi (White et al., 1990). Each PCR reaction contained 1 µl of DNA template of air samples and mock community, 1 U of Taq DNA recombinant polymerase (Promega Corporation, Madison, WI, USA), 1 × PCR reaction buffer, 1.5 mM MgCl_2_, 1 mM of each primer and 0.2 mM dNTPs in a final volume of 25 µL. A GeneAmp PCR system 9700 thermal cycler (Fisher Scientific) was used for amplification. DNA was denatured at 94 °C for 5 min, followed by 35 cycles at 94 °C for 30 s, 48 °C for 30 s, 72 °C for 1 min and elongation at 72 °C for 10 min. The amount of amplicon was visually estimated after gel electrophoresis. A visible smear of PCR products at approximately 340–760 bp was removed and purified using a QIAquick Gel Extraction Kit (QIAGEN, GmbH, Hilden, Germany).

### Bioinformatics analysis

Illumina MiSeq-sequenced paired-end fastq sequences of fungal spores in air samples and the mock community that were amplified using ITS1 and ITS2 barcodes were demultiplexed and barcodes removed by Eurofins Genomics, similar to previous studies (Naveed et al., 2016; Nicolaisen et al., 2017; Rossmann et al., 2021; Senés-Guerrero & Schüßler, 2016). Primers were removed from demultiplexed sequences using the Cutadapt program (Martin, 2011). Reads were quality filtered, denoised, truncated and merged using the clustering-free Divisive Amplicon Denoising Algorithm (DADA2) v.1.14 (Callahan et al., 2016), similar to previous studies (Aguayo et al., 2018; Banchi et al., 2018; Banchi et al., 2020a; Kumari et al., 2016; Mbareche et al., 2020; Nicolaisen et al., 2017; Nilsson et al., 2019b; Schiro et al., 2019; Tordoni et al., 2021). The forward and reverse reads were truncated at 260 bp and 200 bp, respectively, to exclude lower quality reads while retaining only reads with fewer than two expected errors. After inference of sequence variation, reads were merged as described in the DADA2 tutorial. Chimeras were identified and removed, and the resulting amplicon sequences were used in subsequent analyses (Callahan et al., 2017). The “assignTaxonomy” function in the DADA2 pipeline was used for assigning amplicon sequence variants (ASVs) to specific sequences in the UNITE fungal database v.8.2 2020-02-04 (Abarenkov et al., 2020b), similar to previous studies (Banchi et al., 2018; da Silva et al., 2021; de Souza et al., 2021; Dyda et al., 2019; Ogaki et al., 2021). UNITE fungal databases, with 35,077 and 71,723 representative sequences, were used for taxonomic assignment of the ITS1 and ITS2 sequences, respectively. The UNITE eukaryotes database v.8.2 2020-02-04 (Abarenkov et al., 2020a) was used for taxonomic assignment in the mock community, similar to previous studies (Heeger et al., 2018; Tedersoo et al., 2018). Similarly, UNITE eukaryotes databases, with 91,074 and 183,678 representative sequences were used for taxonomic assignment of ITS1 and ITS2 sequences, respectively.

R packages “Phyloseq” v.1.3.0 (McMurdie & Holmes, 2013) and “ggplot2” v.3.3.2 alongside package “Biostrings” v.2.54.0 were used for downstream analysis of the ASV output from DADA2. The resulting taxonomic table was combined with the OTU table, matrix of ASV sequences and sample metadata. The relative abundance of fungal phyla detected using ITS1 and ITS2 barcodes at each site was visualised using bar charts. The data for the *Alternaria* genus and its species were extracted from the total fungal community data. ASVs specific to *Alternaria* species in the air samples and fungal and plant species in the mock community were assigned to the taxonomic groups and bar plots were constructed to visualise their relative abundance. Shannon and Simpson indices (*α*-diversity) combined with abundance were used to measure the richness and diversity of *Alternaria* species within the air samples (Spellerberg & Fedor, 2003). Mann–Whitney *U* test was performed to assess the significance of the α-diversity measures in the air samples using the R package “DESeq2” v.1.26.0, similar to Mbareche et al. (2020), Yang et al. (2018) and Archer et al. (2020). Beta diversity and species composition were assessed using the Bray–Curtis dissimilarity metric whereas UniFrac distance was used to measure phylogenetic community distance in the air samples (Mbareche et al., 2018). Weighted UniFrac and Unweighted UniFrac distances were used to evaluate the phylogenetic distances based on species abundance and presence or absence of the species in each sample, respectively (d’Entremont et al., 2020; Mbareche et al., 2018).

Principal Coordinates Analysis (PCoA) was performed using packages “plyr” v.1.8.6 alongside “ggplot2” to visualise the relationships. The PCoA was separated according to ITS barcodes and the environment of sampling (rural, urban and unmanaged) to examine the clusters. The clusters observed in the PCoA were statistically validated with a permutational analysis of variance (PERMANOVA) test at 999 permutations, similar to previous studies (Banchi et al., 2020a, 2020b; Fort et al., 2016; Tordoni et al., 2021). PERMANOVA was performed using the R package “vegan” v.2.5-6 with the function “adonis” (Oksanen et al., 2019). A *p *value of ≤ 0.05 was considered statistically significant for the Mann–Whitney *U* and PERMANOVA tests. The Mann–Whitney *U* test *p* values were adjusted using the Holm method.

The UNITE database, e.g. v.8.2 2020-02-04 dedicated to bioinformatics pipelines comprises sequences that are annotated, are released within a specific period of time and remain static, whereas the web-based version of the same database is an interactive and up-to-date version whose sequences continuously undergo taxonomic re-annotation to reflect the most recent nomenclatural and taxonomic changes and their associated metadata (Nilsson et al., 2019a). Furthermore, errors that accumulate during sample preparation and sequencing cannot be completely filtered during bioinformatics pipeline annotation of sequences using the static UNITE databases (Anslan et al., 2018; Nilsson et al., 2019b). Therefore, to minimise errors and improve taxonomic affiliations, sequences of all the ASVs of ITS1 and ITS2 were BLAST searched in the web-based UNITE reference database (https://unite.ut.ee/), similar to de Vere et al. (2012), Nilsson et al. (2016) and Anslan et al. (2018). To improve taxonomic specificity, similar to Be et al. (2015), BLAST search results with “*Alternaria*” in the taxon were further analysed to extract their species hypotheses while excluding those without “*Alternaria”* in their taxonomic name. Bioinformatics analyses were performed in R software v.3.6.3 (R Core Team, 2020).

### Meteorological data

Two Campbell Scientific meteorological stations were established at Worcester and Lakeside Circle to provide half-hourly meteorological data for the period July 2017–October 2019. The meteorological stations were co-located with the Burkard 7-day and Cyclone samplers at Worcester and Lakeside Circle. The station at Lakeside Circle provided meteorological data for both itself and the Lakeside Container since they are closely located (310 m). There was a gap in meteorological data from January 2016 to July 2017 before the acquisition of the Campbell Scientific meteorological instruments. The gap in data was filled with hourly meteorological data obtained from a nearby (20 km away) UK Met station (Pershore Weather Station; MET Office, UK). This was after verifying that some of the Pershore weather data had a high correlation with Lakeside and Worcester weather data, similar to the approach of Skjøth et al. (2016).

The half-hourly and hourly weather data were independently averaged to provide daily meteorological data for each meteorological station to match the daily observation of *Alternaria* spores. Selected meteorological parameters including air temperature, pressure, relative humidity, solar radiation, precipitation, wind speed, wind direction, leaf wetness and dew point were extracted for analysis. Spearman’s rank correlation test was performed between the daily *Alternaria* spore concentrations and their corresponding meteorological variables, similar to Grinn-Gofroń et al. (2019) and Olsen et al. (2019).

## Results

### Taxonomy, relative abundance and diversity of *Alternaria* species in air samples

Ten composite air samples with DNA each representing the sampling period of 5 July 2016–09 October 2019 collected at Worcester, Lakeside Container and Lakeside Circle were sequenced. Sequencing, using ITS1 and ITS2 primers, resulted in a total of 926,319 and 644,016 reads after quality filtering, respectively (Table S3). Each sample contained between 56,100 and 116,955 reads for ITS1 and between 42,587 and 76,342 reads for ITS2. Reads were assembled into a total of 12,725 and 5369 ASVs for ITS1 and ITS2, respectively. A total of 114 (out of 1200 sequences) and 126 (out of 2795 sequences) chimeras were identified in ITS1 and ITS2 barcodes, respectively, and removed from the sequences as stipulated for PCR artefacts in the DADA2 guidelines.

### Shared and unique taxa and their abundance

**Fig. 2:**
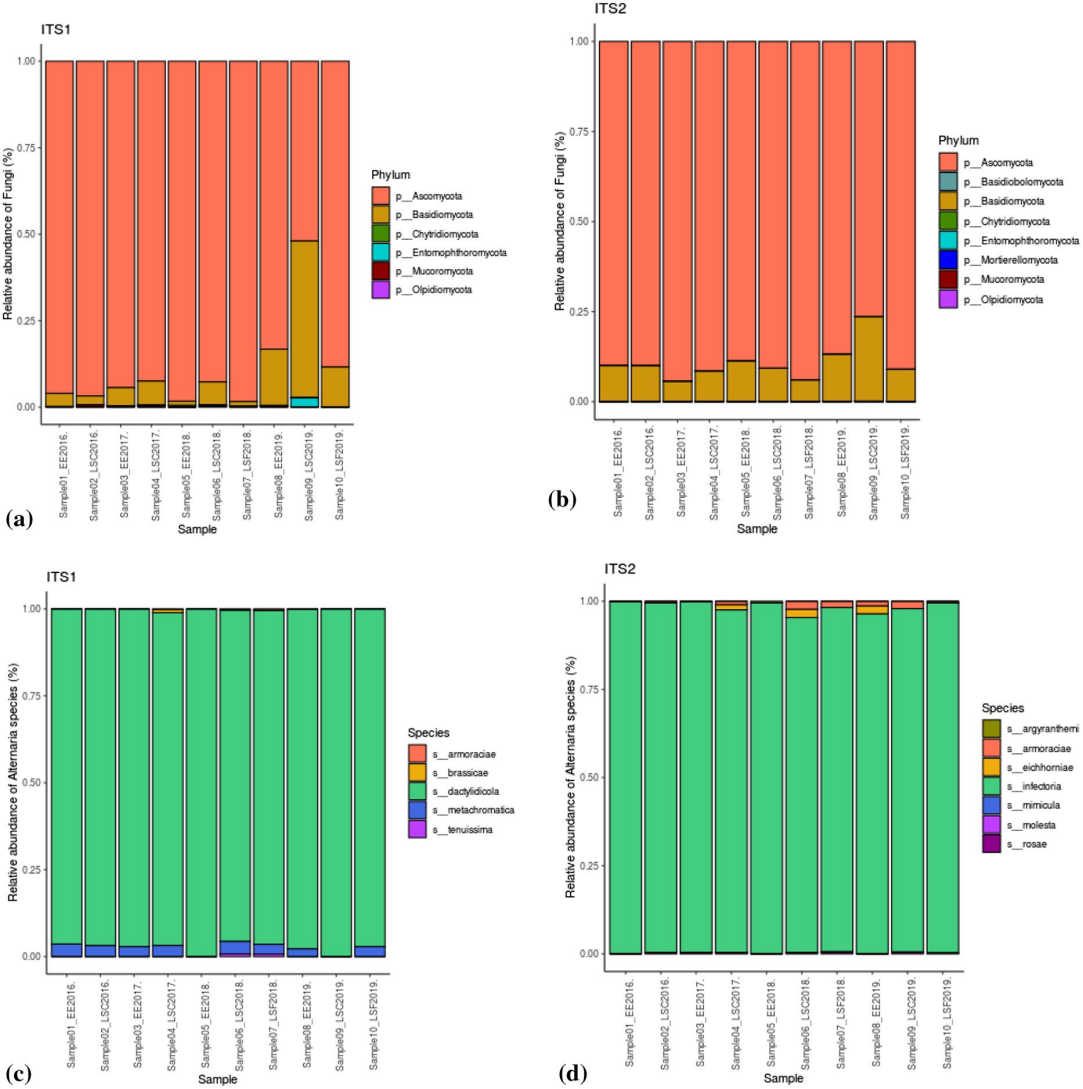
Relative abundance of the different fungal phyla and species of *Alternaria* fungus detected with (**a**, **c**) ITS1 and (**b**, **d**) ITS2 barcodes at Worcester (EE), Lakeside Container (LSC) and Lakeside Circle (LSF) in the period 2016–2019

ITS1 and ITS2 barcodes were notably similar in composition and richness of fungal phyla, i.e. all the phyla detected in ITS1 were also found in ITS2 (Fig. 2a, b). Ascomycota and Basidiomycota were the most abundant phyla in all the sites. *Alternaria* sequences were detected in both ITS1 and ITS2 barcodes and they were correctly identified as Fungi (kingdom), Ascomycota (phylum), Dothideomycetes (class), Pleosporales (order), Pleosporaceae (family) and *Alternaria* (genus). The ITS1 barcode identified *A. brassicae, A. dactylidicola, A. metachromatica, A. armoraciae* and *A. tenuissima* species in the air samples (Fig. 2c). Meanwhile, the ITS2 barcode identified *A. argyranthemi, A. infectoria, A. eichhorniae, A. mimicula, A. molesta, A. armoraciae* and *A. rosae* species (Fig. 2d).

**Fig. 3:**
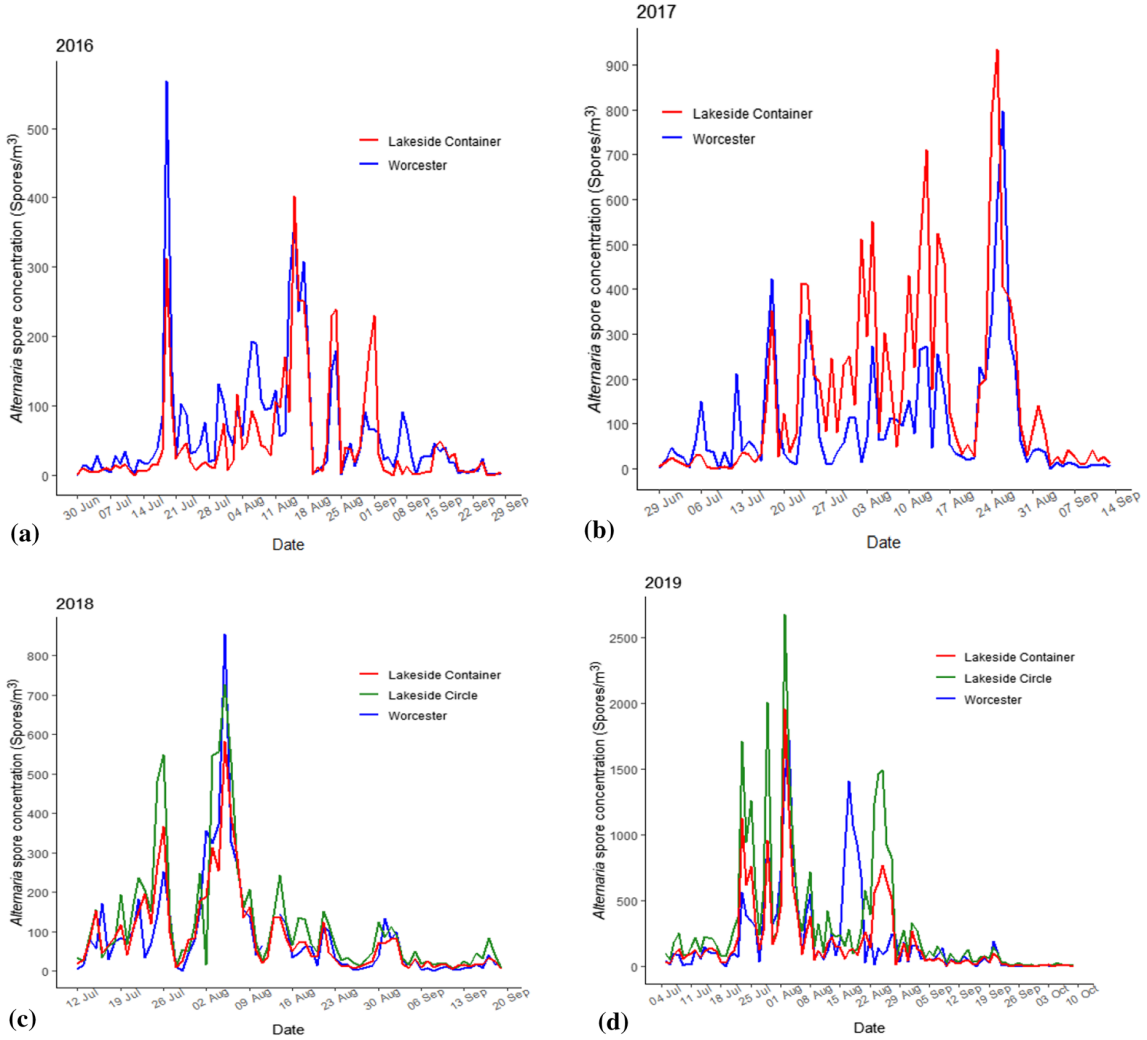
Daily mean *Alternaria* spp. spore concentrations at genus level during the sampling periods of (**a**) 30 Jun–28 Sep 2016, (**b**) 29 Jun–13 Sep 2017, (**c**) 12 Jul–19 Sep 2018 and (**d**) 05 Jul–09 Oct 2019 at Lakeside Container (red lines), Lakeside Circle (green lines) and Worcester (blue lines). Note: no spore sampling at Lakeside Circle in 2016 and 2017 and therefore *Alternaria* spore data for Lakeside Circle are not represented in the time series for 2016 and 2017

**Fig. 4:**
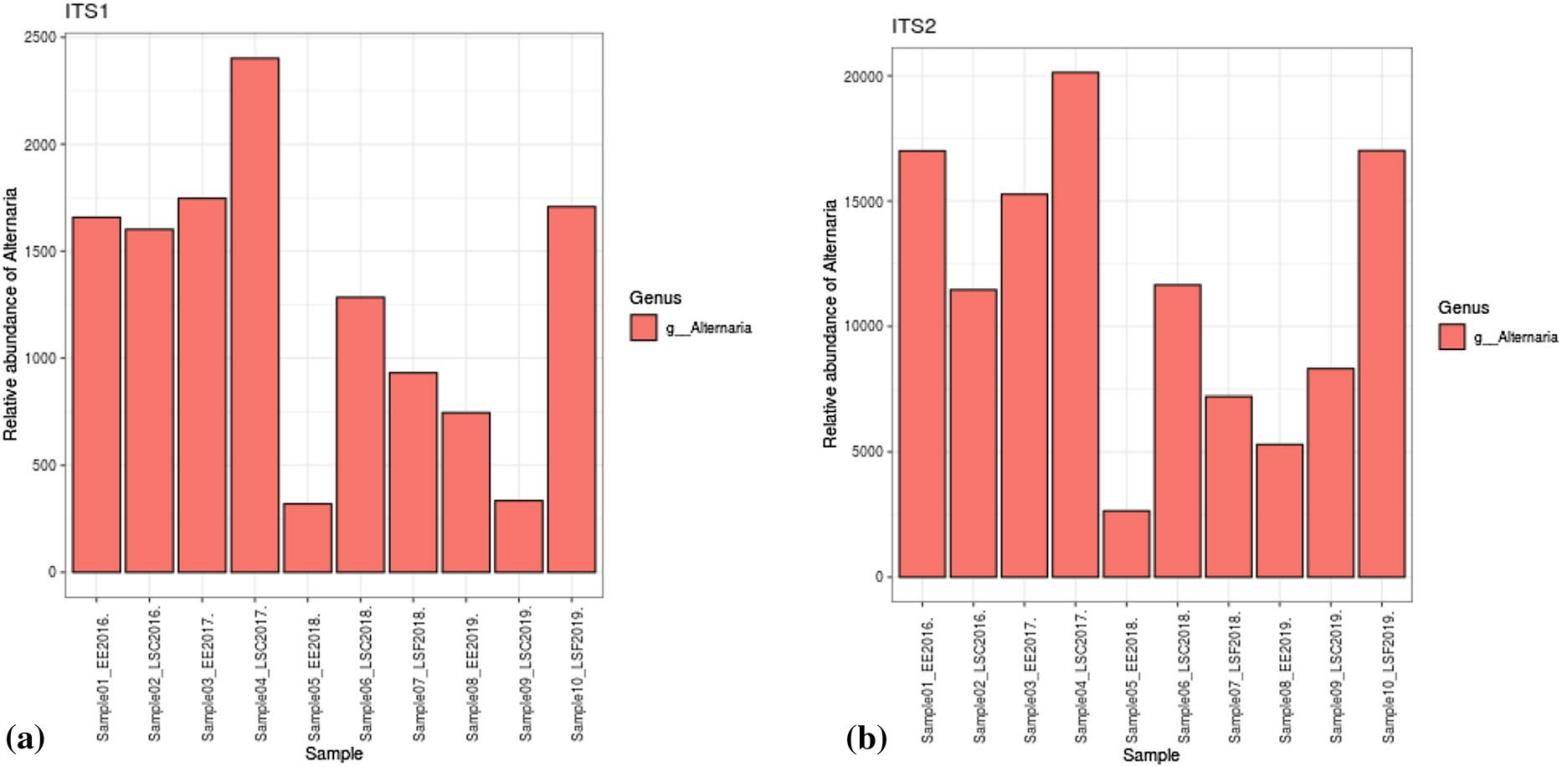
Relative abundance of *Alternaria* spp. at genus level detected with (**a**) ITS1 and (**b**) ITS2 at Worcester (EE), Lakeside Container (LSC) and Lakeside Circle (LSF) during the sampling period of 2016–2019

At the genus level, both the optical microscopy (Fig. 3a–d) and metabarcoding (using ITS1 and ITS2 barcodes in the DADA2 bioinformatics pipeline and UNITE v.8.2 2020-02-04; Fig. 4a, b) approaches corroborated the high abundance of *Alternaria* during the different years of observation. Both methods showed that Worcester had the highest frequency of *Alternaria* in 2016. Meanwhile, Lakeside Container and Lakeside Circle dominated in 2017 and 2019, respectively. However, the approaches differed in 2018 during which the optical microscopy detected the highest frequency of *Alternaria* at Worcester while metabarcoding (using both ITS1 and ITS2 barcodes in the DADA2 bioinformatics pipeline and UNITE v.8.2 2020-02-04) found the maximum at Lakeside Container. High spore concentrations (> 100 spores/m^3^) were observed in July and August of every season. At the species level, according to the ITS1 barcode, *A. dactylidicola* and *A. metachromatica* and for ITS2, *A. infectoria,* were the most abundant *Alternaria* species in the air of Lakeside Container, Lakeside Circle and Worcester. ITS1 barcode showed that *A. dactylidicola* was most abundant at Worcester in 2018 and Lakeside Container in 2019. According to ITS2, *A. infectoria* was the most abundant species at all the sampling sites and years of observation. There was a greater abundance of *A. infectoria* at both Worcester and Lakeside Circle in 2019 compared to 2018.

BLAST search identified 66.67% of the *Alternaria* species that were detected with the DADA2 bioinformatics pipeline. The BLAST search and threshold distance analysis of species hypothesis found *A. metachromatica*, *A. brassicae, A. infectoria, A. eichhorniae A. sonchi, A. planifunda, A. carotiincultae, A. abundans* and other unidentified *Alternaria* species in ITS1 sequences [(Table S4a (i) and (ii)]. *A. infectoria* was the most abundant species (69.6%) detected by the BLAST search.

For ITS2 sequences, the BLAST search and species hypothesis found *A. infectoria, A. armoraciae, A. eichhorniae, A. rosae* and *A. mimicula.* Other species detected by the search included *A. alternata*, *A. metachromatica, A. abundans, A. linicola, A. brassicae, A. triticina, A. solani, A. brassicicola* and other unidentified *Alternaria* species [(Table S4b (i) & (ii)]. *Alternaria* sp. was the most abundant (27.3%), followed by *A. infectoria* (9.1%) and *A. solani* (9.1%) in the BLAST search. Noteworthy, other uncommon *Alternaria* species including *A. oregonensis, A. venezuelensis, A. tropica, A. multirostrata, A. cichorii, A. tumida, A. photistica,* etc. were detected in the BLAST search.

### *Alternaria* species richness and diversity

Alpha diversity was analysed using Shannon and Simpson diversity indices (Table 1). Statistical analysis (Mann–Whitney *U* test) showed significant (Shannon: *p* = 0.01437 and Simpson: *p* = 0.01418) difference in *Alternaria* species richness and diversity in the air samples. In general, the α-diversity showed high diversity and richness of *Alternaria* species at the observation sites. Both Shannon and Simpson indices of ITS1 and ITS2 barcodes showed that Worcester and Lakeside Container were the most and least diverse sites, respectively. Overall, ITS1 and ITS2 barcodes detected five and seven *Alternaria* species, respectively, and Shannon and Simpson indices were highly correlated.

**Table 1:** Alpha diversity analysis output measuring species richness and diversity within the samples using Shannon and Simpson diversity indices

Sample	Location	Environment	Shannon		Simpson	
			ITS1	ITS2	ITS1	ITS2
Sample01_EE2016	Worcester	Urban	1.43	0.85	0.69	0.52
Sample02_LSC2016	Lakeside Container	Rural	1.48	0.83	0.72	0.52
Sample03_EE2017	Worcester	Urban	1.39	0.90	0.68	0.54
Sample04_LSC2017	Lakeside Container	Rural	1.51	0.95	0.72	0.54
Sample05_EE2018	Worcester	Urban	1.04	1.30	0.63	0.67
Sample06_LSC2018	Lakeside Container	Rural	1.53	0.89	0.73	0.54
Sample07_LSF2018	Lakeside Circle	Natural	1.49	0.91	0.71	0.54
Sample08_EE2019	Worcester	Urban	1.58	0.94	0.73	0.55
Sample09_LSC2019	Lakeside Container	Rural	0.84	0.93	0.51	0.55
Sample10_LSF2019	Lakeside Circle	Natural	1.56	0.86	0.73	0.53

The numbers represent mean values for each sample and ITS barcode

**Fig. 5:**
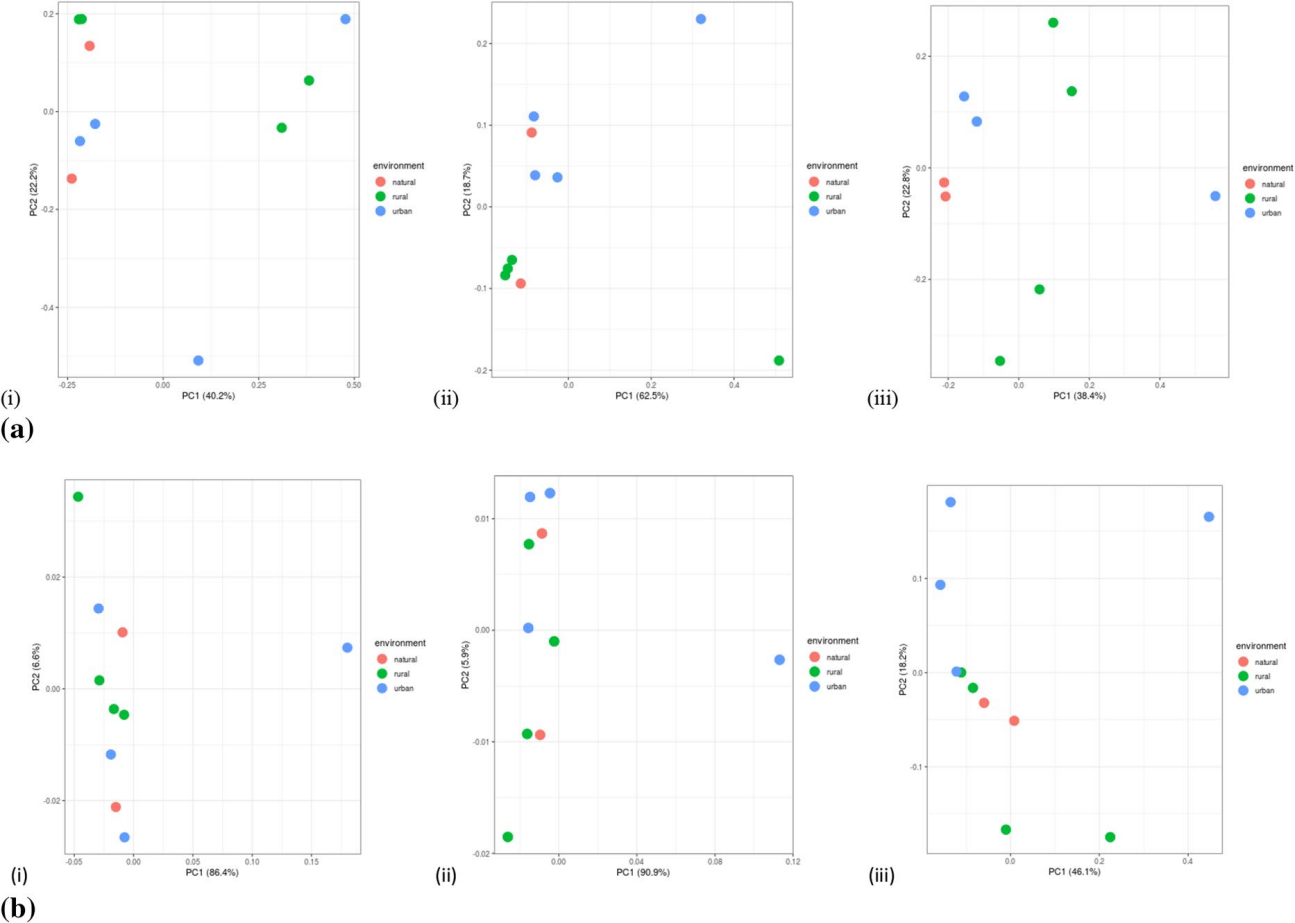
Principal Coordinates Analysis (PCoA) plots of air samples collected from Worcester (urban; blue), Lakeside Container (rural; green) and the unmanaged grassland of Lakeside Circle (natural; red) for the period 2016–2019, generated from ASV sequences of ITS1 (**a**) and ITS2 (**b**) and represented by (i) Bray–Curtis dissimilarity matrix (ii) weighted UniFrac and (iii) unweighted UniFrac distances. The points on the PCoA plots represent the *β*-diversity of *Alternaria* species at both the location of each sample and the whole sampling period (2016–2019)

Beta diversity was assessed using the Bray–Curtis dissimilarity metric, Weighted UniFrac and Unweighted UniFrac distances and visualised using PCoA plots. The maximum percentage variation of β-diversity for ITS1 and ITS2 barcodes was explained by Principal Coordinate (PC1; Fig. 5a, b). Bray–Curtis, weighted UniFrac and unweighted UniFrac metrics showed that air samples from Lakeside Container grouped with those from Worcester and Lakeside Circle. PERMANOVA analysis showed that the observed clusters were not significant (Table 2).

**Table 2:** Variation (*R*^2^) explained by each variable and their interactions using ITS1 and ITS2 barcodes for weighted UniFrac, unweighted UniFrac and Bray–Curtis distances matrices

Variable	Weighted UniFrac		Unweighted UniFrac		Bray–Curtis	
	*R*^2^	*P* value	*R*^2^	*P* value	*R*^2^	*P* value
*ITS1*						
Environment	0.18	0.664	0.27	0.209	0.22	0.406
Year	0.21	0.938	0.13	1.000	0.17	0.786
Season	0.11	0.345	0.06	0.91	0.10	0.54
Environment:Year	0.52	1.000	0.52	1.000	0.37	1.000
Environment:Season	0.43	0.118	0.30	0.207	0.22	0.49
Year:Season	N/A	N/A	N/A	N/A	N/A	N/A
*ITS2*						
Environment	0.18	0.54	0.18	0.724	0.17	0.599
Year	0.24	0.856	0.31	0.598	0.24	0.841
Season	0.03	0.817	0.03	0.982	0.05	0.713
Environment:year	0.54	1.000	0.46	1.000	0.55	1.000
Environment:season	0.11	0.468	0.24	0.534	0.14	0.461
Year:season	N/A	N/A	N/A	N/A	N/A	N/A

*P < 0.05; **P < 0.01; ***P < 0.001

N/A inadequate data points for permutational analysis

### Taxonomy and abundance of mock community individuals

Overall, the plants (except *Corylus*) and the fungi (*Alternaria* and *Cladosporium*) in the mock community were correctly identified from kingdom, phylum, class, order and family to genus level (Table S5; Fig. S1a–d). *Dactylis, Phleum, Cladosporium* and *Alternaria* were the most abundant genera. At the species level, *D. glomerata* (with both ITS1 and ITS2), *P. pratense* (with ITS1), *Q. ilex* and *Q. robur* (both with ITS2) were correctly identified using the UNITE eukaryotes database in the mock community (Fig. S2a and b). Among the plant species, *D. glomerata* and *P. pratense* were the most abundant in the mock community individuals (Fig. S2a and b). Among the fungal species, *A. tenuissima, C. delicatulum* and *C. tenuissimum* were the most abundant species detected (Fig. S2c & d).

However, there were some identification inaccuracies at the species level (Fig. S2a–d). For instance, *A. glutinosa* was identified as *A. firma* (with UNITE eukaryotes database and ITS1) and *A. fauriei* (with UNITE eukaryotes database and ITS2)*, Alternaria* sp. and *A. alternata* were identified as *A. tenuissima* (with both UNITE eukaryotes and UNITE fungal databases and ITS1) and *A. eichhorniae* (with UNITE eukaryotes database and ITS2), *Cladosporium* sp. was identified as *C. delicatulum* (with both UNITE eukaryotes and fungal databases and ITS1) and *C. tenuissimum* (with both UNITE eukaryotes and fungal databases and ITS2), *L. perenne* was identified as *L. temulentum* (with UNITE eukaryotes database and ITS1 and ITS2). *A. vulgaris, B. pendula, C. avenella, U. dioica, P. x hispanica* were not detected in the mock community.

Other unexpected genera were detected in low abundances in the mock community and these included *Aniselyton, Brassica, Chenopodium, Festuca, Laportea*, *Fraxinus* and *Mycocentrospora, Aureobasidium, Botrytis, Calloria, Camarosporium, Coniozyma, Filobasidium, Golovinomyces, Itersonilia, Lichtheimia, Muriphaeosphaeria, Phaecoccomyces, Pringsheimia, Sporobolomyces, Trebouxia* and *Vishniacozyma* among others*.* Similarly, other unexpected species were detected in the mock community and these included *Deutzia gracilis, Europiella artemisiae, Eschscholzia californica, Cochliobolus victoriae* and *Aureobasidium pullulans* among others.

### Effect of meteorological variables on daily *Alternaria* spp. spore concentrations

Spearman’s correlation test showed that several meteorological variables significantly correlated with the daily *Alternaria* spp. spore concentrations at the different sampling sites and seasons (Table 3). Temperature, relative humidity, precipitation and solar radiation were all strongly correlated with the daily spore concentration. Meanwhile, pressure, leaf wetness, wind speed and direction correlated weakly to moderately with the daily *Alternaria* spore concentration. The effect of the individual meteorological variables on the daily spore concentrations varied with observation site and year.

**Table 3:** Spearman’s correlation coefficients between daily mean *Alternaria* spp. spore concentration and weather parameters at Lakeside Container, Lakeside Circle and Worcester for the period 29 Jun–30 Sep 2016, 22 Jun–30 Sep 2017, 22 Jun–30 Sep 2018 and 06 Jul–09 10 Oct 2019

Variables	2016		2017		2018			2019		
	Lakeside container	Worcester	Lakeside container	Worcester	Lakeside container	Lakeside circle	Worcester	Lakeside container	Lakeside circle	Worcester
Wind direction (°)	0.051	− 0.041	− 0.221	0.032	**0.291**	0.082	**0.241**	0.038	0.019	0.015
Wind speed (m/s)	− 0.189	** − 0.213**	− 0.033	** − 0.211**	** − 0.205**	** − 0.367**	** − 0.269**	− 0.153	− 0.133	− 0.036
Pressure (hPa)	**0.290**	**0.225**	0.127	**0.289**	** − 0.292**	− 0.031	** − 0.212**	0.186	0.173	0.094
Temperature (°C)	**0.408**	**0.470**	0.172	**0.511**	**0.337**	**0.613**	**0.467**	**0.661**	**0.661**	**0.468**
Dew point (°C)	0.050	0.090	0.009	0.108	0.451	N/A	**0.391**	N/A	N/A	N/A
Precipitation (mm)	** − 0.460**	** − 0.462**	− 0.131	** − 0.276**	− 0.126	** − 0.359**	** − 0.218**	− **0.376**	− **0.341**	− **0.354**
Relative humidity (%)	** − 0.530**	** − 0.549**	− 0.167	** − 0.506**	** − 0.036**	** − 0.473**	** − 0.219**	− **0.477**	− **0.451**	− **0.479**
Solar radiation (W/m^2^)	N/A	N/A	**0.391**	**0.609**	0.270	**0.604**	**0.368**	**0.534**	**0.532**	**0.521**
Leaf wetness (min)	N/A	N/A	** − 0.282**	** − 0.477**	− 0.069	** − 0.247**	** − 0.237**	0.128	0.106	− 0.173

Significance level *p* < 0.05 (values in bold)

N/A no available data for statistical test

## Discussion

### Diversity and composition of *Alternaria* species in the rural, urban and unmanaged grassland habitats

The optical microscopy and metabarcoding approach showed a high spore concentration and relative abundance of *Alternaria,* respectively, for the sampling sites and in the different years of observation (Figs. 3a–d and 4a, b). Metabarcoding results showed a diversity of *Alternaria* species at Worcester and Lakeside (Fig. 2c, d). The species detected (with ITS1) included *A. dactylidicola, A. metachromatica, A. brassicae, A. armoraciae* and *A. tenuissima* and those with ITS2 included *A. infectoria, A. armoraciae, A. eichhorniae, A. mimicula, A. molesta* and *A. rosae*. BLAST search [Table S4a (i) and b (i)] and species hypotheses [Table S4a (ii) and b (ii)] confirmed 66.67% of the above species detected with the bioinformatics pipeline including *A. alternata*. Meanwhile, the BLAST search did not detect 33.33% of the *Alternaria* species because they could not meet the filtering criteria (similarity score = 93–100%, *E*-value = 0.001) and hence were considered putative artefacts or non-*Alternaria* species (Anslan et al., 2018). This is the first study to reveal the diversity and richness of *Alternaria* species in three heterogeneously diverse biogeographic locations that are ~ 7 km apart. Previous studies had found a high abundance (but not diversity) of *Alternaria* spp. in Worcester (O’Connor et al., 2014; Sadyś et al., 2015; Skjøth et al., 2016). Fungal diversity in an area depends on the pedology, land use, vegetation and climatic conditions of the area to provide substrate and conducive environments for the fungi to grow and multiply (Peay & Bruns, 2014). The Shannon and Simpson indices showed a significant difference in the diversity of *Alternaria* species within the samples. However, Bray–Curtis dissimilarity, Weighted UniFrac and Unweighted UniFrac distance metrics showed low variation in diversity of *Alternaria* species between the sites (Fig. 5a, b). This suggests similarity in *Alternaria* species at the three sites and this could be attributed to the fact that the unmanaged (Lakeside Circle), rural (Lakeside Container) and urban (Worcester) sites share similar vegetation (grass species) and agricultural landscapes since they are only ~ 7 km apart (Apangu et al., 2020; Brennan et al., 2019). Furthermore, the similarity in species diversity could also be attributed to the fact that the airborne *Alternaria* spores undergo dilution and mixing in the lower boundary layer before they are captured at the traps thus contributing to the genetic mixing, species diversity and the final concentration (Fröhlich-Nowoisky et al., 2016; Nicolaisen et al., 2017; Sicard et al., 2016). Moreover, the PCoA showed that the *Alternaria* spp. at the 3 study sites clustered together (Fig. 5a, b), suggesting that the sites share similar *Alternaria* species. Similar studies also found low fungal diversity, richness and composition in airborne samples collected from nearby (< 10 km apart) sites (Abrego et al., 2018; Dannemiller et al., 2014). Contrarily, Mhuireach et al. (2016), with more closely located samplers (50 m apart), reported high variations in abundance and diversity of bacterial and fungal communities. Such fine-scale (< 50 m) variations in species diversity and abundance of bioaerosols can be attributed to weather, season, land use, land cover and distance from bioaerosol sources (Mhuireach et al., 2016; Rathnayake et al., 2016; Rolph et al., 2018). Conversely and expectedly, studies with distantly (> 50 km apart) located sites reported a high fungal diversity due to the effect and interactions of site-specific factors, e.g. vegetation type, changing atmospheric conditions and shifts in microbial sources (Banchi et al., 2018; Bowers et al., 2013; Fröhlich-Nowoisky et al., 2012). Nonetheless, sometimes locations (even though distantly apart) may not have strong effects or require interaction with other variables to influence the diversity of fungal communities. For instance, Nicolaisen et al. (2017) found similar results to ours in that sampling location alone did not considerably contribute to airborne fungal diversity except between seasons and their interactions. Our study only presents the diversity and abundance of *Alternaria* species in the summer and early autumn and found no significant effect of season or their interaction with sampling site on *Alternaria* spp. diversity (Table 2). This is attributed to the fact that Worcester and Lakeside are neighbouring sites that likely have a synchronised *Alternaria* spore season in the middle of summer, as was previously reported in Central-Northern Europe (Skjøth et al., 2016). However, Banchi et al. (2020a) and Kumari et al. (2016) examined the influence of location, season and their interactions on fungal diversity and found that their interactions (especially during summer) greatly influenced fungal diversity. Meanwhile, Mbareche et al. (2020) considered only location and found that samples collected from sites of compost, biomethanisation and dairy farms significantly influenced fungal diversity in those areas. Overall, this study suggests that regardless of an urban, rural or unmanaged location, areas that border each other and have similar land use and land cover types tend to have low *Alternaria* species diversity and similar species composition. However, since we only investigated the *Alternaria* genus, further studies are needed to examine fungal diversity for a whole fungal community in such areas to draw a firm conclusion.

*A. dactylidicola* and *A. metachromatica* were among the *Alternaria* species detected in the air samples. *A. dactylidicola* and *A. metachromatica* are species observed for the first time in Worcester and Lakeside, the UK and they are relatively new species. Simmons (1994) first morphologically described *A. metachromatica* as a pathogenic species and together with *A. infectoria* belongs to the *Alternaria* section *Infectoriae* (Woudenberg et al., 2013). *A. dactylidicola* is a grass-inhabiting species that was first found to associate with the leaves, roots and dead stems of the grass *D. glomerata* in Italy and it is phylogenetically linked with *Alternaria cesenica* (Thambugala et al., 2017). *Lolium*, *Holcus* and *Dactylis* are the most abundant grass genera in Worcester (including Lakeside) and are harvested as feeds for livestock (Brennan et al., 2019; Frisk et al., 2021). Harvesting of grasses for hay is a common practice among livestock farmers and starts from June to September and peaks in July (Jefferson, 2005). Several studies have shown that cutting/mowing of grasses either for hay or in public parks releases a considerable amount of *Alternaria* spores into the atmosphere (Astray et al., 2010; Comtois et al., 1995; Corden et al., 2003; Irga & Torpy, 2016; Kilic et al., 2010; Mitakakis et al., 2001). Moreover, increased *Alternaria* spore concentrations in public urban parks were observed in July and August (Kasprzyk et al., 2021), a period when high *Alternaria* spp. spore concentrations were recorded in Worcester and Lakeside (Fig. 3) and when the parks are intensively utilised. Apart from grass mowing for hay, Worcester urban green areas such as grasslands, pastureland, public parks, the racecourse and the cricket ground are potential sources and were previously associated with *Alternaria* spores in the city (Sadyś et al., 2016). In Worcester, the grass species, *D. glomerata,* flowers in June–August (Brennan et al., 2019; Frisk et al., 2021), which coincides with the period of high *Alternaria* spp. spore concentrations in Worcester (Apangu et al., 2020). The *Alternaria* in the grasses can also actively or passively release many spores into the atmosphere when weather conditions are optimal (Crandall & Gilbert, 2017). Several meteorological parameters including temperature, humidity, rain, wind speed and direction significantly correlated with *Alternaria* spp. spore concentrations at the three sites (Table 3). Therefore, the high abundance of *A. dactylidicola, A. metachromatica* and *A. infectoria* in all the study sites could be attributed to the favourable weather conditions and the abundance of *Dactylis* grass in Worcester and Lakeside since it is a major host to the species (Brennan et al., 2019; Sadyś et al., 2015). Przemieniecki et al. (2019) found that endophytic fungi, including *Alternaria,* were capable of colonising multiple grass taxa as was observed with *A. alternata* inhabiting both *L. perenne* and *P. pratense*. Previously, *Alternaria* sp. was also found in the leaves and roots of the grass *Holcus lanatus* (Márquez et al., 2010). We hypothesise that *A*. *dactylidicola* can also colonise *Lolium* and *Holcus* grasses since those grass genera are abundant in Worcester (including Lakeside) (Brennan et al., 2019).

*A. dactylidicola* is a saprobe (Thambugala et al., 2017), suggesting that it is active in decomposition within unmanaged grasslands and nutrient recycling in terrestrial ecosystems. Whereas pollen from *D. glomerata*, the main host of *A*. *dactylidicola,* is highly allergenic (D’Amato et al., 1991; Frisk et al., 2021), it is unknown whether *A*. *dactylidicola* also has such allergenic attributes since it is a newly discovered species. Neither is there any study on the pathological properties of *A. dactylidicola.* Future studies should elucidate the pathogenic and allergenic capabilities of *A. dactylidicola.* The high abundance of *A. dactylidicola* in the different sampling sites and years could also be related to differences in local meteorological conditions as it was found that local weather, e.g. temperature, relative humidity, rain, solar radiation, etc. observed at Worcester, Lakeside Container and Lakeside Circle all correlated with the daily *Alternaria* spore concentrations. Recently, Grinn-Gofroń et al. (2018) and Tordoni et al. (2021) observed a strong effect of the local weather and climate on fungal sporulation and the eventual release of the spores into the atmosphere.

The abundance of *A. metachromatica* in Worcester is linked to its host, oilseed rape (*Brassica napus*) (Al-lami et al., 2020). Oilseed rape is abundantly grown around Worcester and Lakeside and high concentrations of *Alternaria* spp. spores in Worcester were associated with its harvesting (Apangu et al., 2020). *A. metachromatica* is a multi-host pathogenic fungus as it was found to cause leaf spot on tomatoes (*Lycopersicon esculentum*) (Bashir et al., 2014) and infected the pasture plant, spotted knapweed (*Centaurea stoebe),* which is native to Europe (Broennimann et al., 2014). Related to *A. metachromatica* are *A. solani*, *A. alternata, A. infectoria, A. arborescens, A. tenuissima, A. mimicula* (Bessadat et al., 2017; Ramezani et al., 2019) and *A. tomatophila* (Rodrigues et al., 2010) that cause early blight in tomatoes and potatoes.

*A. metachromatica* belongs to the *A. infectoria* species group that is associated with many grass taxa in the family Poaceae, including wheat, barley, oat and rye (Andersen et al., 2002). Wheat, barley and oat are widely grown in Worcestershire and their combined harvesting was associated with high concentrations of *Alternaria* spp. spores in the area (Apangu et al., 2020). It is, therefore, likely that the harvesting of wheat, barley, oat and ryegrass (for hay) could have contributed to the high abundance of *A. metachromatica* in Worcester and Lakeside.

*Alternaria* spp. data of 2018 and 2019 analysed from Lakeside Circle strongly indicated that *Alternaria* spores were being released from Lakeside Circle when the grasses were still green and not expected to host *Alternaria*, suggesting that the litter from previous seasons was hosting *Alternaria*. Daily spore data analysis also showed that *Alternaria* spp. spore concentrations at Worcester and Lakeside Circle sites increased drastically from 2018 to 2019 (Fig. 3c, d). Metabarcoding results showed that there was a gradual increase in the abundance of *A. dactylidicola, A. metachromatica* and *A. infectoria* at Worcester and Lakeside Circle from 2018 to 2019 (Fig. 2c). Therefore, the increases in the abundance of *A. dactylidicola, A. metachromatica* and *A. infectoria* could also be attributed to the accumulation (2017–2019) of litter at the unmanaged site (Lakeside Circle) and other unmanaged sites surrounding Worcester and Lakeside, which provide a habitable environment for the species above. Similarly, de Vries et al. (2007) found that fungal biomass increased with the age of vegetation and they attributed it to the accumulation of organic matter content and minimal disturbance from agronomic practices, e.g. tillage, which encouraged the growth of the mycelial network. The current study presents the abundance and diversity of *Alternaria* species for the whole sampling period. Future studies should investigate species diversity and abundance at much higher temporal resolutions, e.g. weekly or three days, similar to Brennan et al. (2019), to produce precise and near real-time information for allergy sufferers, crop pathologists and medical practitioners.

Other *Alternaria* species detected by the ITS1 barcode included *A. armoraciae*, *A. brassicae* and *A. tenuissima*. *A. armoraciae* and *A. tenuissima* belong to the sections *Chalastospora* and *Alternata*, respectively, meanwhile the section of *A. brassicae* is yet undefined (Woudenberg et al., 2013). Like *A. metachromatica, A. armoraciae* and *A. brassicae* are species that infect plants in the *Brassicaceae* family, e.g. *Brassica oleracea* (da Cruz Cabral et al., 2016) and *B. napus* (Al-lami et al., 2020). Meanwhile, like *A. dactylidicola, A. tenuissima* infects crops and grasses in the Poaceae family including wheat and *Dactylis* (Dang et al., 2015; Thambugala et al., 2017). Other species detected by the ITS2 barcode included *A. argyranthemi, A. armoraciae*, *A. eichhorniae*, *A. mimicula*, *A. molesta* and *A. rosae* (Fig. 2d)*.* BLAST search and threshold distance of species hypotheses also confirmed that *A. alternata*, *A. armoraciae, A. eichhorniae, A. rosae* and *A. mimicula* were among the *Alternaria* species in the air of Worcester, Lakeside Container and Lakeside Circle (Table S4b ii). *A. alternata* and *A. eichhorniae* belong to the section *Alternata* while *A. mimicula* and *A. molesta* belong to sections *Brassicicola* and *Phragmosporae*, respectively, and *A. argyranthemi* remains undefined (Woudenberg et al., 2013). The BLAST search also detected *A. solani* in the air samples. *A. solani* causes early blight in tomatoes and potatoes and therefore could have originated from the intensive horticultural farms and allotments (Agriculture & Horticulture Development Board, 2015; Apangu et al., 2020). Although the intensive farms manage the early blight through fungicide applications at earlier growth stages, such applications do not affect *Alternaria* sporulation at maturing and senescence stage of the plants (Skjøth et al., 2012). *A. eichhorniae* was detected at all the three sites and in all years of observation but was most abundant at Worcester in 2018. *A. eichhorniae* infects water hyacinth (*Eichhornia crassipes*) (Shabana et al., 2001) and parasitises other species of water hyacinth such as *E. azurea, E. diversifolia, E. heterosperma, E. natans* which are phylogenetically related (Cook, 1998; Pellegrini et al., 2018). Although *E. crassipes* is native to the Amazon basin, it was introduced as an ornamental plant (and grown in ponds) in more than 50 countries including the UK before it was banned by the European Union in 2016 (Patoka et al., 2016). Cases of allergy related to *Alternaria* and other allergens were previously reported in florist shops in Worcester (Emberlin et al., 2004). The detection of *A. eichhorniae* in the air samples suggests that *E. crassipes* weed still exists in ponds, lakes and rivers around Worcester and Lakeside and *A. eichhorniae* spores were possibly passively dispersed by the wind from such sources. Lakeside sampling site has two nearby Lakes (Fig. 1) and several ponds while River Severn (Fig. 1) flows through Worcester, which can all harbour *E. crassipes*, the host of *A. eichhorniae.* Studies have shown that thousands of invasive *Eichhornia* species including *E****.**** crassipes* have been introduced to freshwater ecosystems worldwide (Johansson et al., 2018; Scriver et al., 2015). Another possibility could be that *A. eichhorniae* spores were transported from other remote sources to the UK, as seen with soybean rust spores being transported for over 1000 km in the USA (Isard et al., 2005, 2007). This also demonstrates the robustness of the DADA2 pipeline in detecting rare species of fungi (Nearing et al., 2018). *A. mimicula* causes early blight in tomato plants *(Lycopersicon esculentum)* (Ramezani et al., 2019; Woudenberg et al., 2013)*. A. molesta* causes skin lesions on Harbour porpoise (*Phocoena phocoena*), a marine mammal that lives in coastal areas and river estuaries (Mamgain et al., 2013; Woudenberg et al., 2013). *P. phocoena*, being a protected cetacean among endangered species in the UK and EU, are abundant in British waters (Roberts et al., 2019). Magyar et al. (2016) explained that, through the “spiralling” process, suspended fungal propagules in flowing waters can be transported some distance (200–1000 m) before settling on leaves or other substrates at riverbanks or streambanks and eventually passively dispersed into the air. Fungal asexual spores of aquatic hyphomycetes have been found to use such dispersal pathways (Duarte et al., 2012; Magyar et al., 2016; Thomas et al., 1991). The River Severn and Bristol Channel are the possible habitats of *P. phocoena* nearest to Worcester and Lakeside. It is, therefore, possible that *P. phocoena* could have shed *A. molesta* spores into the river, which are later deposited at the riverbanks by spring bore tides and eventually passively dispersed into the air and detected at Worcester and Lakeside. Apart from seawater and seawater animals, e.g. *P. phocoena*, strains of *A. molesta* have also been found in soils and seawater plants but not land plants (Lawrence et al., 2016). *A. rosae* is a species in the section *Pseudoalternaria* (Lawrence et al., 2016). It causes black head mould in wheat and barley and infects sweet briar (*Rosa rubiginosa*) rose plants (Poursafar et al., 2018). *A. rosae* is also associated with locoweeds *Astragalus variabilis* and *Sphaerophysa salsula* that are commonly found in grazing grasslands and are toxic to animals (Lu et al., 2017). Overall, *A. infectoria, A. metachromatica, A. brassicae, A. eichhorniae, A. mimicula, A. rosae*, *A. armoraciae* and *A. alternata* were the main pathogenic *Alternaria* species detected in the air of Worcester, Lakeside Container and Lakeside Circle. *A. infectoria* and *A. metachromatica* were the most abundant pathogenic species*.*

### Allergenic *Alternaria* species

The allergenicity of *Alternaria* spores to sensitised individuals is well documented (D’Amato et al., 1997). High cases of prescriptions for allergic rhinitis and asthma-related hospital admissions were previously reported from the population living in Worcester (including Lakeside) in the past (Emberlin & Lewis, 2006; Rowney et al., 2021; Watson et al., 1996). Phylogenetic analysis of *Alternaria* spores based on Alt a 1 allergen gene sequence shows that *A. tenuissima* is closely related with *A. alternata* while *A. metachromatica* is closely linked with *A. infectoria* and all are allergenic species (Hong et al., 2005). Other allergenic *Alternaria* species in this study include *A. mimicula* and *A. argyranthemi* (Hong et al., 2005). *A. rosae* causes cutaneous infection in humans (Liu et al., 2017). Meanwhile, no study has investigated the allergenic properties in *A*. *dactylidicola, A. eichhorniae*, *A. molesta* and *A. armoraciae.*

### Similarities and differences between ITS1 and ITS2 barcodes

Although the ITS1 and ITS2 barcodes had similar fungal composition and richness at phylum level and UNITE database correctly identified all the fungi (and plants) in the air samples and mock community from kingdom to genus level (Table S5; Fig. S1a–d), there were notable variations at the species level (Table S5; Fig. S2a–d). For instance, *A. alternata* was incorrectly identified as *A. tenuissima* but correctly identified by BLAST search. Moreover, the ITS1 barcode differed from the ITS2 barcode in *Alternaria* species identification, apart from *A. armoraciae.* Such misidentifications and barcode differences are attributed to the quality of the reference databases and the biases and artefacts associated with the bioinformatics pipelines and these were also previously recognised (Abrego et al., 2018; Aguayo et al., 2021; Pauvert et al., 2019; Piper et al., 2019; Vasar et al., 2021). However, the BLAST search of the ASV sequences was performed to identify the exact taxonomy of the sequences and minimise the errors from the bioinformatics pipeline. The misidentifications in *Alternaria* spp. are also partly attributed to the several taxonomic re-descriptions of the 275 known *Alternaria* species, e.g. 32 new combinations, 16 new species and 10 old names resurrected, resulting in the addition of new species and transfer of some species to other genera, e.g. *Prathoda* (Simmons, 2007; Woudenberg et al., 2013). *Alternaria* morphospecies (300), indistinguishable using multi-gene phylogeny due to environmental conditions, are synonymised under *A. alternata* (He et al., 2021; Woudenberg et al. (2015)*,* suggesting that some of the *Alternaria* species closely related with *A. alternata* could have been identified as other related species or as *A. alternata.* These findings suggest that taxonomic resolution of *Alternaria* species remains to be fully addressed for a more accurate molecular identification of *Alternaria* species in the future. de Vere et al. (2012) and Bulman et al. (2018) also recognised the uncertainty in species discrimination and emphasised that correct species identification of microorganisms is largely dependent on the quality of sequences in the reference databases.

## Conclusion

The results revealed several *Alternaria* species detected in the air of Worcester and Lakeside. These included *A. dactylidicola, A. metachromatica, A. brassicae, A. tenuissima* and *A. armoraciae* from ITS1 barcoding. Meanwhile, ITS2 barcoding revealed *A. infectoria, A. alternata*, *A. argyranthemi, A. armoraciae, A. molesta, A. mimicula, A. rosae* and *A. eichhorniae.* Some of the species, e.g. *A. dactylidicola, A. metachromatica, A. armoraciae*, *A. argyranthemi, A. armoraciae, A. molesta, A. mimicula, A. rosae* and *A. eichhorniae* are uncommon species detected for the first time in Worcester and Lakeside. *A. dactylidicola* and *A. metachromatica* were highly abundant in Worcester and Lakeside. The BLAST search and species hypothesis confirmed 66.67% of the above species (including *A. alternata*). Whereas the three sites exhibited a significant species diversity at each site (Shannon and Simpson indices), there was a minimum difference (PERMANOVA) in species diversity between the sites due to the uniformity of habitats in the three sites, the proximity of the sites to each other and genetic mix of the airborne spores before they are captured at the traps.

## Supplementary Information

Below is the link to the electronic supplementary material.

Supplementary file1 (PDF 282 kb)

## References

[CR1] Abarenkov, K., Zirk, A., Piirmann, T., Pöhönen, R., Ivanov, F., Nilsson, R. H. & Kõljalg, U. (2020b). *UNITE general FASTA release for Fungi. Version 04.02.2020b**. UNITE Community.*

[CR2] Abarenkov, K., Zirk, A., Piirmann, T., Pöhönen, R., Ivanov, F., Nilsson, R. H. & Kõljalg, U. (2020a). *UNITE general FASTA release for Eukaryotes. Version 04.02.2020a**. UNITE Community*.

[CR3] Abrego N, Norros V, Halme P, Somervuo P, Ali-Kovero H, Ovaskainen O (2018). Give me a sample of air and I will tell which species are found from your region: Molecular identification of fungi from airborne spore samples. Molecular Ecology Resources.

[CR4] Adams-Groom B, Emberlin J, Corden J, Millington W, Mullins J (2002). Predicting the start of the birch pollen season at London, Derby and Cardiff, United Kingdom, using a multiple regression model, based on data from 1987 to 1997. Aerobiologia.

[CR5] Agriculture and Horticulture Development Board. (2015). *GB end-November stocks up 23% on previous season*. https://potatoes.ahdb.org.uk/publications/gb-end-november-stocks-23-previous-season

[CR6] Aguayo J, Fourrier-Jeandel C, Husson C, Loos R (2018). Assessment of Passive Traps Combined with High-Throughput. Applied and Environmental Microbiology.

[CR7] Aguayo J, Husson C, Chancerel E, Fabreguettes O, Chandelier A, Fourrier-Jeandel C, Dupuy N, Dutech C, Ioos R, Robin C, Thibaudon M, Marçais B, Desprez-Loustau ML (2021). Combining permanent aerobiological networks and molecular analyses for large-scale surveillance of forest fungal pathogens: A proof-of-concept. Plant Pathology.

[CR9] Al-lami HFD, You MP, Mohammed AE, Barbetti MJ (2020). Virulence variability across the *Alternaria* spp. population determines incidence and severity of Alternaria leaf spot on rapeseed. Plant Pathology.

[CR10] Andersen B, Krøger E, Roberts RG (2002). Chemical and morphological segregation of *Alternaria arborescens*, *A. infectoria* and *A. tenuissima* species-groups. Mycological Research.

[CR11] Anslan S, Nilsson RH, Wurzbacher C, Baldrian P, Tedersoo L, Bahram M (2018). Great differences in performance and outcome of high-throughput sequencing data analysis platforms for fungal metabarcoding. MycoKeys.

[CR12] Apangu GP, Frisk CA, Adams-Groom B, Satchwell J, Pashley CH, Skjøth CA (2020). Air mass trajectories and land cover map reveal cereals and oilseed rape as major local sources of *Alternaria* spores in the Midlands, UK. Atmospheric Pollution Research.

[CR13] Archer SDJ, Lee KC, Caruso T, King-Miaow K, Harvey M, Huang D, Wainwright BJ, Pointing SB (2020). Air mass source determines airborne microbial diversity at the ocean–atmosphere interface of the Great Barrier Reef marine ecosystem. ISME Journal.

[CR14] Astray G, Rodríguez-Rajo FJ, Ferreiro-Lage JA, Fernández-González M, Jato V, Mejuto JC (2010). The use of artificial neural networks to forecast biological atmospheric allergens or pathogens only as *Alternaria* spores. Journal of Environmental Monitoring.

[CR15] Awad AHA (2005). Vegetation: A source of air fungal bio-contaminant. Aerobiologia.

[CR16] BAF (1995). Airborne pollens and spores: A guide to trapping and counting.

[CR17] Banchi E, Ametrano CG, Greco S, Stanković D, Muggia L, Pallavicini A (2020). PLANiTS: A curated sequence reference dataset for plant ITS DNA metabarcoding. Database.

[CR18] Banchi E, Ametrano CG, Stanković D, Verardo P, Moretti O, Gabrielli F, Lazzarin S, Borney MF, Tassan F, Tretiach M, Pallavicini A, Muggia L (2018). DNA metabarcoding uncovers fungal diversity of mixed airborne samples in Italy. PLoS ONE.

[CR19] Banchi E, Ametrano CG, Tordoni E, Stanković D, Ongaro S, Tretiach M, Pallavicini A, Muggia L, Verardo P, Tassan F, Trobiani N, Moretti O, Borney MF, Lazzarin S (2020). Environmental DNA assessment of airborne plant and fungal seasonal diversity. Science of the Total Environment.

[CR21] Bashir U, Mushtaq S, Akhtar N (2014). First report of *Alternaria metachromatica* from Pakistan causing leaf spot of tomato. Pakistan Journal of Agricultural Sciences.

[CR22] Be NA, Thissen JB, Fofanov VY, Allen JE, Rojas M, Golovko G, Fofanov Y, Koshinsky H, Jaing CJ (2015). Metagenomic analysis of the airborne environment in urban spaces. Microbial Ecology.

[CR23] Bessadat N, Berruyer R, Hamon B, Bataille-Simoneau N, Benichou S, Kihal M, Henni DE, Simoneau P (2017). *Alternaria* species associated with early blight epidemics on tomato and other Solanaceae crops in northwestern Algeria. European Journal of Plant Pathology.

[CR24] Bowers RM, Clements N, Emerson JB, Wiedinmyer C, Hannigan MP, Fierer N (2013). Seasonal variability in bacterial and fungal diversity of the near-surface atmosphere. Environmental Science and Technology.

[CR26] Brennan GL, Potter C, de Vere N, Griffith GW, Skjøth CA, Osborne NJ, Wheeler BW, McInnes RN, Clewlow Y, Barber A, Hanlon HM, Hegarty M, Jones L, Kurganskiy A, Rowney FM, Armitage C, Adams-Groom B, Ford CR, Petch GM, The PollerGEN Consortium and Creer, S. (2019). Temperate airborne grass pollen defined by spatio-temporal shifts in community composition. Nature Ecology and Evolution.

[CR27] Broennimann O, Mráz P, Petitpierre B, Guisan A, Müller-Schärer H (2014). Contrasting spatio-temporal climatic niche dynamics during the Eastern and Western invasions of spotted knapweed in North America. Journal of Biogeography.

[CR28] Bulman SR, McDougal RL, Hill K, Lear G (2018). Opportunities and limitations for DNA metabarcoding in Australasian plant-pathogen biosecurity. Australasian Plant Pathology.

[CR29] Callahan BJ, McMurdie PJ, Holmes SP (2017). Exact sequence variants should replace operational taxonomic units in marker-gene data analysis. ISME Journal.

[CR30] Callahan BJ, McMurdie PJ, Rosen MJ, Han AW, Johnson AJA, Holmes SP (2016). DADA2: High-resolution sample inference from Illumina amplicon data. Nature Methods.

[CR31] Chen L, Fang K, Dong XF, Yang AL, Li YX, Zhang HB (2020). Characterization of the fungal community in the canopy air of the invasive plant *Ageratina adenophora* and its potential to cause plant diseases. PLoS ONE.

[CR32] Clasen LA, Detheridge AP, Scullion J, Griffith GW (2020). Soil stabilisation for DNA metabarcoding of plants and fungi. Implications for sampling at remote locations or via third-parties. Metabarcoding and Metagenomics.

[CR34] Comtois P, Morand S, Infante-Rivard C, Gautrin D, Vanderplass O, Malo JL (1995). Exposure to spores during mowing: A comparative assessment of workers, parks and town. Aerobiologia.

[CR35] Cook CDK, Kubitzki K (1998). Pontederiaceae. Flowering plants. Monocotyledons. The families and genera of vascular Plants.

[CR36] Corden JM, Millington WM, Mullins J (2003). Long-term trends and regional variation in the aeroallergen *Alternaria* in Cardiff and Derby UK—are differences in climate and cereal production having an effect?. Aerobiologia.

[CR37] Crandall SG, Gilbert GS (2017). Meteorological factors associated with abundance of airborne fungal spores over natural vegetation. Atmospheric Environment.

[CR38] D’Amato G, Chatzigeorgiou G, Corsico R, Gioulekas D, Jäger L, Jäger S, Kontou-Fili K, Kouridakis S, Liccardi G, Meriggi A, Palma-Carlos A, Palma-Carlos ML, Pagan Aleman A, Parmiani S, Puccinelli P, Russo M, Spieksma FT, Torricelli R, Wüthrich B (1997). Evaluation of the prevalence of skin prick test positivity to *Alternaria* and *Cladosporium* in patients with suspected respiratory allergy. A European multicenter study promoted by the Subcommittee on Aerobiology and Environmental Aspects of Inhalant Allerg. Allergy.

[CR39] D’Amato G, De Palma R, Verga A, Martucci P, Liccardi G, Lobefalo G (1991). Antigenic activity of nonpollen parts (leaves and stems) of allergenic plants (*Parietaria judaica *and* Dactylis glomerata*). Annals of Allergy, Asthma & Immunology.

[CR40] d’Entremont TW, Migicovsky Z, López-Gutiérrez JC, Walker AK (2020). Saltmarsh rhizosphere fungal communities vary by sediment type and dominant plant species cover in Nova Scotia, Canada. Environmental Microbiology Reports.

[CR41] da Cruz Cabral L, Terminiello L, Fernández Pinto V, Fog Nielsen K, Patriarca A (2016). Natural occurrence of mycotoxins and toxigenic capacity of *Alternaria* strains from mouldy peppers. International Journal of Food Microbiology.

[CR42] da Silva TH, Câmara PEAS, Pinto OHB, Carvalho-Silva M, Oliveira FS, Convey P, Rosa CA, Rosa LH (2021). Diversity of fungi present in permafrost in the South Shetland Islands, Maritime Antarctic. Microbial Ecology.

[CR43] Dang HX, Pryor B, Peever T, Lawrence CB (2015). The *Alternaria* genomes database: A comprehensive resource for a fungal genus comprised of saprophytes, plant pathogens, and allergenic species. BMC Genomics.

[CR44] Dannemiller KC, Mendell MJ, Macher JM, Kumagai K, Bradman A, Holland N, Harley K, Eskenazi B, Peccia J (2014). Next-generation DNA sequencing reveals that low fungal diversity in house dust is associated with childhood asthma development. Indoor Air.

[CR45] de Souza LMD, Ogaki MB, Câmara PEAS, Pinto OHB, Convey P, Carvalho-Silva M, Rosa CA, Rosa LH (2021). Assessment of fungal diversity present in lakes of Maritime Antarctica using DNA metabarcoding: A temporal microcosm experiment. Extremophiles.

[CR46] de Vere N, Rich TCG, Ford CR, Trinder SA, Long C, Moore CW, Satterthwaite D, Davies H, Allainguillaume J, Ronca S, Tatarinova T, Garbett H, Walker K, Wilkinson MJ (2012). DNA barcoding the native flowering plants and conifers of wales. PLoS ONE.

[CR47] de Vries FT, Bloem J, van Eekeren N, Brusaard L, Hoffland E (2007). Fungal biomass in pastures increases with age and reduced N input. Soil Biology and Biochemistry.

[CR48] Degois J, Simon X, Bontemps C, Leblond P, Duquenne P (2019). Characterization of experimental complex fungal bioaerosols: Impact of analytical method on fungal composition measurements. Aerosol Science and Technology.

[CR49] Duarte S, Seena S, Bärlocher F, Cássio F, Pascoal C (2012). Preliminary insights into the phylogeography of six aquatic hyphomycete species. PLoS ONE.

[CR50] Dyda M, Pyzik A, Wilkojc E, Kwiatkowska-Kopka B, Sklodowska A (2019). Bacterial and fungal diversity inside the medieval building constructed with sandstone plates and lime mortar as an example of the microbial colonization of a nutrient-limited extreme environment (Wawel Royal Castle, Krakow, Poland). Microorganisms.

[CR51] Emberlin J, Adamsgroom B, Treu R, Carswell F (2004). Airborne pollen and fungal spores in Florist shops in Worcester and in Bristol, UK: A potential problem for occupational health. Aerobiologia.

[CR52] Emberlin JC, Lewis RA (2006). A double blind, placebo controlled trial of inert cellulose powder for the relief of symptoms of hay fever in adults. Current Medical Research and Opinion.

[CR53] Ettenauer JD, Piñar G, Lopandic K, Spangl B, Ellersdorfer G, Voitl C, Sterflinger K (2012). Microbes on building materials—Evaluation of DNA extraction protocols as common basis for molecular analysis. Science of the Total Environment.

[CR54] Fort T, Robin C, Capdevielle X, Delière L, Vacher C (2016). Foliar fungal communities strongly differ between habitat patches in a landscape mosaic. PeerJ.

[CR55] Frisk CA, Adams-groom B, Skjøth CA (2021). Stochastic flowering phenology in *Dactylis glomerata* populations described by Markov chain modelling. Aerobiologia.

[CR56] Fröhlich-Nowoisky J, Burrows SM, Xie Z, Engling G, Solomon PA, Fraser MP, Mayol-Bracero OL, Artaxo P, Begerow D, Conrad R, Andreae MO, Després VR, Pöschl U (2012). Biogeography in the air: Fungal diversity over land and oceans. Biogeosciences.

[CR57] Fröhlich-Nowoisky J, Kampf CJ, Weber B, Huffman JA, Pöhlker C, Andreae MO, Lang-Yona N, Burrows SM, Gunthe SS, Elbert W, Su H, Hoor P, Thines E, Hoffmann T, Després VR, Pöschl U (2016). Bioaerosols in the Earth system: Climate, health, and ecosystem interactions. Atmospheric Research.

[CR58] Gabriel MF, Postigo I, Gutiérrez-Rodríguez A, Suñén E, Tomaz CT, Martínez J (2015). Development of a PCR-based tool for detecting immunologically relevant Alt a 1 and Alt a 1 homologue coding sequences. Medical Mycology.

[CR59] Galán C, Smith M, Thibaudon M, Frenguelli G, Oteros J, Gehrig R, Berger U, Clot B, Brandao R (2014). Pollen monitoring: Minimum requirements and reproducibility of analysis. Aerobiologia.

[CR60] Grinn-Gofroń A, Bosiacka B, Bednarz A, Wolski T (2018). A comparative study of hourly and daily relationships between selected meteorological parameters and airborne fungal spore composition. Aerobiologia.

[CR61] Grinn-Gofroń A, Nowosad J, Bosiacka B, Camacho I, Pashley C, Belmonte J, De Linares C, Ianovici N, Manzano JMM, Sadyś M, Skjøth C, Rodinkova V, Tormo-Molina R, Vokou D, Fernández-Rodríguez S, Damialis A (2019). Airborne *Alternaria* and *Cladosporium* fungal spores in Europe: Forecasting possibilities and relationships with meteorological parameters. Science of the Total Environment.

[CR201] He L, Cheng H, Zhao L, Htun AA, Yu ZH, Deng JX, Li QL (2021). Morphological and molecular identification of two new Alternaria species (Ascomycota, Pleosporaceae) in section Radicina from China. MycoKeys.

[CR62] Hebert PDN, Cywinska A, Ball SL, DeWaard JR (2003). Biological identifications through DNA barcodes. Proceedings of the Royal Society B: Biological Sciences.

[CR63] Heeger F, Bourne EC, Baschien C, Yurkov A, Bunk B, Spröer C, Overmann J, Mazzoni CJ, Monaghan MT (2018). Long-read DNA metabarcoding of ribosomal RNA in the analysis of fungi from aquatic environments. Molecular Ecology Resources.

[CR64] Hirst JM (1952). An automatic volumetric spore trap. Annals of Applied Biology.

[CR65] Hong SG, Cramer RA, Lawrence CB, Pryor BM (2005). Alt a 1 allergen homologs from *Alternaria* and related taxa: Analysis of phylogenetic content and secondary structure. Fungal Genetics and Biology.

[CR66] Irga PJ, Torpy FR (2016). A survey of the aeromycota of Sydney and its correspondence with environmental conditions: Grass as a component of urban forestry could be a major determinant. Aerobiologia.

[CR67] Isard SA, Gage SH, Comtois P, Russo JM (2005). Principles of the atmospheric pathway for invasive species applied to soybean rust. BioScience.

[CR68] Isard SA, Russo JM, Ariatti A (2007). The integrated aerobiology modeling system applied to the spread of soybean rust into the Ohio River valley during September 2006. Aerobiologia.

[CR69] Jefferson RG (2005). The conservation management of upland hay meadows in Britain: A review. Grass and Forage Science.

[CR70] Johansson ML, Dufour BA, Wellband KW, Corkum LD, MacIsaac HJ, Heath DD (2018). Human-mediated and natural dispersal of an invasive fish in the eastern Great Lakes. Heredity.

[CR71] Joly M, Peuch VH (2012). Objective classification of air quality monitoring sites over Europe. Atmospheric Environment.

[CR72] Kasprzyk I (2008). Non-native Ambrosia pollen in the atmosphere of Rzesz (SE Poland); Evaluation of the effect of weather conditions on daily concentrations and starting dates of the pollen season. International Journal of Biometeorology.

[CR73] Kasprzyk I, Grinn-Gofroń A, Ćwik A, Kluska K, Cariñanos P, Wójcik T (2021). Allergenic fungal spores in the air of urban parks. Aerobiologia.

[CR74] Kilic M, Ufuk Altintas D, Yilmaz M, Güneşer Kendirli S, Bingöl Karakoc G, Taskin E, Ceter T, Pinar NM (2010). The effects of meteorological factors and *Alternaria* spore concentrations on children sensitised to *Alternaria*. Allergologia Et Immunopathologia.

[CR75] Kumari P, Woo C, Yamamoto N, Choi HL (2016). Variations in abundance, diversity and community composition of airborne fungi in swine houses across seasons. Scientific Reports.

[CR76] Lacey J, Allit U (1995). Airborne pollen and spores: A guide to trapping and counting.

[CR200] Lee HB, Patriarca A, Magan N (2015). Alternaria in food: Ecophysiology, mycotoxin production and toxicology. Mycobiology.

[CR77] Lawrence DP, Rotondo F, Gannibal PB (2016). Biodiversity and taxonomy of the pleomorphic genus *Alternaria*. Mycological Progress.

[CR78] Leppänen HK, Täubel M, Jayaprakash B, Vepsäläinen A, Pasanen P, Hyvärinen A (2018). Quantitative assessment of microbes from samples of indoor air and dust. Journal of Exposure Science and Environmental Epidemiology.

[CR79] Leuschner C, Gebel S, Rose L (2013). Root trait responses of six temperate grassland species to intensive mowing and NPK fertilisation: A field study in a temperate grassland. Plant and Soil.

[CR80] Lin WR, Wang PH, Tien CJ, Chen WY, Yu YA, Hsu LY (2018). Changes in airborne fungal flora along an urban to rural gradient. Journal of Aerosol Science.

[CR81] Liu AW, Bateman AC, Greenbaum A, Garvin K, Clarridge J, Grim J (2017). Cutaneous phaeohyphomycosis in a hematopoietic stem cell transplant patient caused by *Alternaria rosae*: First case report. Transplant Infectious Disease.

[CR82] Lu H, Haiyun Q, Qiwu Z, Zhenhui R, Ruixu X, Baoyu Z, Rebecca C (2017). Endogenous fungi isolated from three locoweed species from rangeland in western China. African Journal of Microbiology Research.

[CR84] Magyar D, Vass M, Li DW, Li DW (2016). Dispersal strategies of microfungi. Biology of microfungi.

[CR86] Mamgain A, Roychowdhury R, Tah J (2013). Review: *Alternaria* pathogenicity and its strategic controls. Research Journal of Biology.

[CR87] Márquez SS, Bills GF, Acuña LD, Zabalgogeazcoa I (2010). Endophytic mycobiota of leaves and roots of the grass *Holcus lanatus*. Fungal Diversity.

[CR89] Martin M (2011). Cutadapt removes adapter sequences from high-throughput sequencing reads. Embnet Journal.

[CR90] Maude RB, Humpherson-Jones FM (1980). Studies on the seed-borne phases of dark leaf spot *Alternaria brassicicola* and grey leaf spot *Alternaria brassicae* of brassicas. Annals of Applied Biology.

[CR91] Mbareche H, Veillette M, Bilodeau GJ, Duchaine C (2018). Bioaerosol sampler choice should consider efficiency and ability of samplers to cover microbial diversity. Applied and Environmental Microbiology.

[CR92] Mbareche H, Veillette M, Bilodeau G, Duchaine C (2020). Comparison of the performance of ITS1 and ITS2 as barcodes in amplicon-based sequencing of bioaerosols. PeerJ.

[CR93] McMurdie PJ, Holmes S (2013). Phyloseq: An R package for reproducible interactive analysis and graphics of microbiome census data. PLoS ONE.

[CR94] Meena M, Gupta SK, Swapnil P, Zehra A, Dubey MK, Upadhyay RS (2017). *Alternaria* toxins: Potential virulence factors and genes related to pathogenesis. Frontiers in Microbiology.

[CR95] Meena M, Swapnil P, Upadhyay RS (2017). Isolation, characterization and toxicological potential of *Alternaria*-mycotoxins (TeA, AOH and AME) in different *Alternaria* species from various regions of India. Scientific Reports.

[CR96] Mhuireach G, Johnson BR, Altrichter AE, Ladau J, Meadow JF, Pollard KS, Green JL (2016). Urban greenness influences airborne bacterial community composition. Science of the Total Environment.

[CR97] Mitakakis TZ, Clift A, McGee PA (2001). The effect of local cropping activities and weather on the airborne concentration of allergenic *Alternaria* spores in rural Australia. Grana.

[CR98] Naveed M, Herath L, Moldrup P, Arthur E, Nicolaisen M, Norgaard T, Ferré TPA, de Jonge LW (2016). Spatial variability of microbial richness and diversity and relationships with soil organic carbon, texture and structure across an agricultural field. Applied Soil Ecology.

[CR99] Nearing JT, Douglas GM, Comeau AM, Langille MGI (2018). Denoising the Denoisers: An independent evaluation of microbiome sequence error- correction approaches. PeerJ.

[CR100] Nicolaisen M, West JS, Sapkota R, Canning GGM, Schoen C, Justesen AF (2017). Fungal communities including plant pathogens in near surface air are similar across northwestern Europe. Frontiers in Microbiology.

[CR101] Nilsson RH, Anslan S, Bahram M, Wurzbacher C, Baldrian P, Tedersoo L (2019). Mycobiome diversity: High-throughput sequencing and identification of fungi. Nature Reviews Microbiology.

[CR102] Nilsson RH, Larsson KH, Taylor AFS, Bengtsson-Palme J, Jeppesen TS, Schigel D, Kennedy P, Picard K, Glöckner FO, Tedersoo L, Saar I, Kõljalg U, Abarenkov K (2019). The UNITE database for molecular identification of fungi: Handling dark taxa and parallel taxonomic classifications. Nucleic Acids Research.

[CR103] Nilsson RH, Wurzbacher C, Bahram M, Coimbra VRM, Larsson E, Tedersoo L, Eriksson J, Ritter CD, Svantesson S, Sánchez-García M, Ryberg M, Kristiansson E, Abarenkov K (2016). Top 50 most wanted fungi. MycoKeys.

[CR104] Nowicki M, Nowakowska M, Niezgoda A, Kozik E (2012). *Alternaria* black spot of Crucifers: Symptoms, importance of disease, and perspectives of resistance breeding. Vegetable Crops Research Bulletin.

[CR105] O’Connor DJ, Sadyś M, Skjøth CA, Healy DA, Kennedy R, Sodeau JR (2014). Atmospheric concentrations of *Alternaria, Cladosporium, Ganoderma* and *Didymella* spores monitored in Cork (Ireland) and Worcester (England) during the summer of 2010. Aerobiologia.

[CR106] Ogaki MB, Câmara PEAS, Pinto OHB, Lirio JM, Coria SH, Vieira R, Carvalho-Silva M, Convey P, Rosa CA, Rosa LH (2021). Diversity of fungal DNA in lake sediments on Vega Island, north-east Antarctic Peninsula assessed using DNA metabarcoding. Extremophiles.

[CR107] Oksanen, J., Blanchet, F. G., Kindt, R., Legendre, P., Minchin, P. R. & O’Hara, R. B. (2019). Community Ecology Package: package “vegan” v2.5–6. In *CRAN* (pp. 1–298).

[CR108] Olsen Y, Gosewinkel UB, Skjøth CA, Hertel O, Rasmussen K, Sigsgaard T (2019). Regional variation in airborne *Alternaria* spore concentrations in Denmark through 2012–2015 seasons: The influence of meteorology and grain harvesting. Aerobiologia.

[CR110] Paldy A, Bobvos J, Fazekas B, Manyoki G, Malnasi T, Magyar D (2014). Characterisation of the pollen season by using climate specific pollen indicators. European Journal of Occupational and Environmental Medicine.

[CR112] Pashley CH, Fairs A, Free RC, Wardlaw AJ (2012). DNA analysis of outdoor air reveals a high degree of fungal diversity, temporal variability, and genera not seen by spore morphology. Fungal Biology.

[CR113] Patoka J, Bláha M, Kalous L, Vrabec V, Buřič M, Kouba A (2016). Potential pest transfer mediated by international ornamental plant trade. Scientific Reports.

[CR114] Pauvert C, Buée M, Laval V, Edel-Hermann V, Fauchery L, Gautier A, Lesur I, Vallance J, Vacher C (2019). Bioinformatics matters: The accuracy of plant and soil fungal community data is highly dependent on the metabarcoding pipeline. Fungal Ecology.

[CR115] Peay KG, Bruns TD (2014). Spore dispersal of basidiomycete fungi at the landscape scale is driven by stochastic and deterministic processes and generates variability in plant-fungal interactions. New Phytologist.

[CR116] Pellegrini MOO, Horn CN, Almeida RF (2018). Total evidence phylogeny of Pontederiaceae (Commelinales) sheds light on the necessity of its recircumscription and synopsis of *Pontederia *L. PhytoKeys.

[CR118] Piper AM, Batovska J, Cogan NOI, Weiss J, Cunningham JP, Rodoni BC, Blacket MJ (2019). Prospects and challenges of implementing DNA metabarcoding for high-throughput insect surveillance. GigaScience.

[CR119] Poursafar A, Ghosta Y, Orina AS, Gannibal PB, Javan-Nikkhah M, Lawrence DP (2018). Taxonomic study on *Alternaria* sections *Infectoriae* and *Pseudoalternaria* associated with black (sooty) head mold of wheat and barley in Iran. Mycological Progress.

[CR120] Przemieniecki SW, Damszel M, Kurowski TP, Mastalerz J, Kotlarz K (2019). Identification, ecological evaluation and phylogenetic analysis of non-symbiotic endophytic fungi colonizing timothy grass and perennial ryegrass grown in adjacent plots. Grass and Forage Science.

[CR121] R Core Team. (2020). *The R foundation for statistical computing platform.* https://www.r-project.org/

[CR122] Ramezani Y, Taheri P, Mamarabadi M (2019). Identification of *Alternaria* spp. associated with tomato early blight in Iran and investigating some of their virulence factors. Journal of Plant Pathology.

[CR123] Rathnayake CM, Metwali N, Baker Z, Jayarathne T, Kostle PA, Thorne PS, Shaughnessy PTO, Stone EA (2016). Urban enhancement of PM_10_ bioaerosol tracers relative to background locations in the Midwestern United States. Journal of Geophysical Research: Atmospheres.

[CR124] Roberts L, Collier S, Law S, Gaion A (2019). The impact of marine vessels on the presence and behaviour of harbour porpoise (*Phocoena phocoena*) in the waters off Berry Head, Brixham (South West England). Ocean and Coastal Management.

[CR125] Rodrigues TTMS, Berbee ML, Simmons EG, Cardoso CR, Reis A, Maffia LA, Mizubuti ESG (2010). First report of *Alternaria tomatophila* and *A. grandis* causing early blight on tomato and potato in Brazil. New Disease Reports.

[CR127] Rolph CA, Gwyther CL, Tyrrel SF, Nasir ZA, Drew GH, Jackson SK, Khera S, Hayes ET, Williams B, Bennett A, Collins S, Walsh K, Kinnersley R, Gladding TL (2018). Sources of airborne endotoxins in ambient air and exposure of nearby communities—A review. Atmosphere.

[CR128] Rosa LH, Pinto OHB, Convey P, Carvalho-Silva M, Rosa CA, Câmara PEAS (2020). DNA metabarcoding to assess the diversity of airborne fungi present over Keller Peninsula, King George Island, Antarctica. Microbial Ecology.

[CR129] Rosa LH, Pinto OHB, Šantl-Temkiv T, Convey P, Carvalho-Silva M, Rosa CA, Câmara PEAS (2020). DNA metabarcoding of fungal diversity in air and snow of Livingston Island, South Shetland Islands, Antarctica. Scientific Reports.

[CR130] Rossmann S, Lysøe E, Skogen M, Talgø V, Brurberg MB (2021). DNA metabarcoding reveals broad presence of plant pathogenic oomycetes in soil from internationally traded plants. Frontiers in Microbiology.

[CR131] Rotem J (1994). The genus alternaria: Biology, epidemiology and pathogenicity.

[CR132] Rowney FM, Brennan GL, Skjøth CA, Wheeler B, Osborne NJ, Creer S (2021). Report Environmental DNA reveals links between abundance and composition of airborne grass pollen and respiratory health Environmental DNA reveals links between abundance and composition of airborne grass pollen and respiratory health. Current Biology.

[CR133] Sadyś M, Adams-Groom B, Herbert RJ, Kennedy R (2016). Comparisons of fungal spore distributions using air sampling at Worcester, England (2006–2010). Aerobiologia.

[CR134] Sadyś M, Skjøth CA, Kennedy R (2014). Back-trajectories show export of airborne fungal spores (*Ganoderma* sp.) from forests to agricultural and urban areas in England. Atmospheric Environment.

[CR135] Sadyś M, Skjøth CA, Kennedy R (2015). Determination of *Alternaria* spp. habitats using 7-day volumetric spore trap, Hybrid Single Particle Lagrangian Integrated Trajectory model and geographic information system. Urban Climate.

[CR136] Sanchez H, Bush RK (2001). A review of *Alternaria alternata* sensitivity. Revista Iberoamericana De Micologia.

[CR137] Schiro G, Colangeli P, Müller MEH (2019). A metabarcoding analysis of the mycobiome of wheat ears across a topographically heterogeneous field. Frontiers in Microbiology.

[CR138] Schoch CL, Seifert KA, Huhndorf S, Robert V, Spouge JL, Levesque CA, Chen W, Bolchacova E, Voigt K, Crous PW, Miller AN, Wingfield MJ, Aime MC, An KD, Bai FY, Barreto RW, Begerow D, Bergeron MJ, Blackwell M (2012). Nuclear ribosomal internal transcribed spacer (ITS) region as a universal DNA barcode marker for Fungi. Proceedings of the National Academy of Sciences of the United States of America.

[CR139] Scriver M, Marinich A, Wilson C, Freeland J (2015). Development of species-specific environmental DNA (eDNA) markers for invasive aquatic plants. Aquatic Botany.

[CR140] Seifert KA, Gams W (2011). The genera of Hyphomycetes—2011 update. Persoonia: Molecular Phylogeny and Evolution of Fungi.

[CR141] Senés-Guerrero C, Schüßler A (2016). A conserved arbuscular mycorrhizal fungal core-species community colonizes potato roots in the Andes. Fungal Diversity.

[CR143] Shabana YM, Elwakil MA, Charudattan R (2001). Effect of nutrition and physical factors on mycelial growth and production of pigments and nonchromatic UV-absorbing compounds of *Alternaria eichhorniae*. Journal of Phytopathology.

[CR144] Sharma A, Clark E, McGlothlin JD, Mittal SK (2015). Efficiency of Airborne Sample Analysis Platform (ASAP) bioaerosol sampler for pathogen detection. Frontiers in Microbiology.

[CR146] Shokere LA, Holden MJ, Ronald Jenkins G (2009). Comparison of fluorometric and spectrophotometric DNA quantification for real-time quantitative PCR of degraded DNA. Food Control.

[CR147] Sicard M, Izquierdo R, Alarcón M, Belmonte J, Comerón A, Baldasano JM (2016). Near-surface and columnar measurements with a micro pulse lidar of atmospheric pollen in Barcelona, Spain. Atmospheric Chemistry and Physics.

[CR148] Simmons EG (2007). Alternaria: An identification manual, vol. 6.

[CR149] Simmons EG (1994). Alternaria themes and variations (106-111). Mycotaxon.

[CR151] Skjøth CA, Baker P, Sadyś M, Adams-Groom B (2015). Pollen from alder (*Alnus* sp.), birch (*Betula* sp.) and oak (*Quercus* sp.) in the UK originate from small woodlands. Urban Climate.

[CR152] Skjøth CA, Damialis A, Belmonte J, De Linares C, Fernández-Rodríguez S, Grinn-Gofroń A, Jędryczka M, Kasprzyk I, Magyar D, Myszkowska D, Oliver G, Páldy A, Pashley CH, Rasmussen K, Satchwell J, Thibaudon M, Tormo-Molina R, Vokou D, Ziemianin M, Werner M (2016). *Alternaria* spores in the air across Europe: Abundance, seasonality and relationships with climate, meteorology and local environment. Aerobiologia.

[CR153] Skjøth CA, Sommer J, Brandt J, Hvidberg M, Geels C, Hansen KM, Hertel O, Frohn LM, Christensen JH (2008). Copenhagen—A significant source of birch (*Betula*) pollen?. International Journal of Biometeorology.

[CR154] Skjøth CA, Sommer J, Frederiksen L, Gosewinkel Karlson U (2012). Crop harvest in Denmark and Central Europe contributes to the local load of airborne *Alternaria* spore concentrations in Copenhagen. Atmospheric Chemistry and Physics.

[CR155] Smith DJ, Jaffe DA, Birmele MN, Griffin DW, Schuerger AC, Hee J, Roberts MS (2012). Free Tropospheric Transport of microorganisms from Asia to North America. Microbial Ecology.

[CR156] Spellerberg IF, Fedor PJ (2003). A tribute to Claude-Shannon (1916–2001) and a plea for more rigorous use of species richness, species diversity and the “Shannon-Wiener” Index. Global Ecology and Biogeography.

[CR157] Tedersoo L, Tooming-Klunderud A, Anslan S (2018). PacBio metabarcoding of Fungi and other eukaryotes: Errors, biases and perspectives. New Phytologist.

[CR158] Thambugala KM, Wanasinghe DN, Phillips AJL, Camporesi E, Bulgakov TS, Phukhamsakda C, Ariyawansa HA, Goonasekara ID, Phookamsak R, Dissanayake A, Tennakoon DS, Tibpromma S, Chen YY, Liu ZY, Hyde KD (2017). Mycosphere notes 1–50: Grass (Poaceae) inhabiting Dothideomycetes. Mycosphere.

[CR159] Thomas K, Chilvers GA, Norris RH (1991). A dynamic model of fungal spora in a freshwater stream. Mycological Research.

[CR160] Tordoni E, Ametrano CG, Banchi E, Ongaro S, Pallavicini A, Bacaro G, Muggia L (2021). Integrated eDNA metabarcoding and morphological analyses assess spatio-temporal patterns of airborne fungal spores. Ecological Indicators.

[CR161] Vasar M, Davison J, Neuenkamp L, Sepp S, Young JPW, Moora M, Öpik M (2021). User-friendly bioinformatics pipeline gDAT (graphical downstream analysis tool) for analysing rDNA sequences. Molecular Ecology Resources.

[CR162] von Mutius E (2008). Rhinitis as predictor of adult-onset asthma. The Lancet.

[CR163] Wady L, Shehabi A, Szponar B, Pehrson C, Sheng Y, Larsson L (2004). Heterogeneity in microbial exposure in schools in Sweden, Poland and Jordan revealed by analysis of chemical markers. Journal of Exposure Analysis and Environmental Epidemiology.

[CR165] Watson JP, Cowen P, Lewis RA (1996). The relationship between asthma admission rates, routes of admission, and socioeconomic deprivation. European Respiratory Journal.

[CR166] White, T. J., Bruns, T., Lee, S. & Taylor, J. (1990). Amplification and direct sequencing of fungal ribosomal RNA genes for phylogenetics: PCR—protocols and applications—a laboratory manual. In *PCR protocols: A guide to methods and applications* (pp. 315–322). Academic Press, Inc.

[CR168] Wilson HE, Carroll GC, Roy BA, Kai Blaisdell G (2014). Tall fescue is a potential spillover reservoir host for *Alternaria* species. Mycologia.

[CR169] Woudenberg JHC, Groenewald JZ, Binder M, Crous PW (2013). *Alternaria* redefined. Studies in Mycology.

[CR170] Woudenberg JHC, Seidl MF, Groenewald JZ, de Vries M, Stielow JB, Thomma BPHJ, Crous PW (2015). *Alternaria* section *Alternaria*: Species, formae speciales or pathotypes?. Studies in Mycology.

[CR171] Yang RH, Su JH, Shang JJ, Wu YY, Li Y, Bao DP, Yao YJ (2018). Evaluation of the ribosomal DNA internal transcribed spacer (ITS), specifically ITS1 and ITS2, for the analysis of fungal diversity by deep sequencing. PLoS ONE.

