# Supplementary material for: Environmental DNA reveals diversity and abundance of Alternaria species in neighbouring heterogeneous landscapes in Worcester, UK
Source: Aerobiologia (Bologna). 2022 Oct 23;38(4):457–81. doi: 10.1007/s10453-022-09760-9 (PMC9715499; doi:10.1007/s10453-022-09760-9)
Supplement: Supplementary file 1 — Supplementary file1 (PDF 282 kb) [file 10453_2022_9760_MOESM1_ESM.pdf]

**Electronic Supplementary material**

**Environmental DNA reveals diversity and abundance of *Alternaria* species in neighbouring heterogeneous landscapes in Worcester, UK**

**Journal: Aerobiologia**

**Godfrey Philliam Apangu[a1*]. Carl Alexander Frisk[a2]. Geoffrey M. Petch[a]. Lucia Muggia[b]. Alberto Pallavicini[b]. Mary Hanson[a]. Carsten Ambelas Skjøth[a3]**

[a]School of Science and the Environment, University of Worcester, Henwick Grove, WR2 6AJ, Worcester, UK.

[b]Department of Life Sciences, University of Trieste, via Giorgieri 10, I-34127 Trieste, Italy

[*]Corresponding author. E-mail: apag1_16@uni.worc.ac.uk

Present address:

[1]Protecting Crops and the Environment, Rothamsted Research, West Common, Harpenden, AL5 2JQ, Hertfordshire, U.K.
Email: godfrey.apangu@rothamsted.ac.uk, Tel: +447960301812
[2]Department of Urban Greening and Vegetation Ecology, Norwegian Institute of Bioeconomy Research, Ås, Norway
[3]Department of Environmental Science, Aarhus University, Frederiksborgvej 399, 4000, Roskilde, Denmark

Table S1. Summary of the daily air samples pooled for DNA extraction

| Collection date (2016) | Pool | Site | Collection date (2017) | Pool | Site |
|---|---|---|---|---|---|
| 21-27 Jul | 1 | Worc & LSC | 06-12 Jul | 1 | Worc & LSC |
| 28 Jul-3 Aug | 2 | Worc & LSC | 13-19 Jul | 2 | Worc & LSC |
| 4-10 Aug | 3 | Worc & LSC | 20-26 Jul | 3 | Worc & LSC |
| 11-17 Aug | 4 | Worc & LSC | 27 Jul-02 Aug | 4 | Worc & LSC |
| 18-24 Aug | 5 | Worc & LSC | 03-09 Aug | 5 | Worc & LSC |
| 25-31 Aug | 6 | Worc & LSC | 10-16 Aug | 6 | Worc & LSC |
| 1-7 Sep | 7 | Worc & LSC | 17-23 Aug | 7 | Worc & LSC |
| 8-14 Sep | 8 | Worc & LSC | 24-30 Aug | 8 | Worc & LSC |
| 15-21 Sep | 9 | Worc & LSC | 31 Aug-06 Sep | 9 | Worc & LSC |
| 22-28 Sep | 10 | Worc & LSC | 07-13 Sep | 10 | Worc & LSC |
| **(2018)** | | | **(2019)** | | |
| 12-18 Jul | 1 | Worc, LSC & LSF | 5-10 Jul | 1 | Worc, LSC & LSF |
| 19-25 Jul | 2 | Worc, LSC & LSF | 11-17 Jul | 2 | Worc, LSC & LSF |
| 26 Jul-01 Aug | 3 | Worc, LSC & LSF | 18-24 Jul | 3 | Worc, LSC & LSF |
| 2-8 Aug | 4 | Worc, LSC & LSF | 25-31 Jul | 4 | Worc, LSC & LSF |
| 9-15 Aug | 5 | Worc, LSC & LSF | 01-07 Aug | 5 | Worc, LSC & LSF |
| 16-22 Aug | 6 | Worc, LSC & LSF | 08-14 Aug | 6 | Worc, LSC & LSF |
| 23-29 Aug | 7 | Worc, LSC & LSF | 15-21 Aug | 7 | Worc, LSC & LSF |
| 30 Aug-05 Sep | 8 | Worc, LSC & LSF | 22-28 Aug | 8 | Worc, LSC & LSF |
| 6-12 Sep | 9 | Worc, LSC & LSF | 29 Aug-04 Sep | 9 | Worc, LSC & LSF |
| 13-19 Sep | 10 | Worc, LSC & LSF | 05-11 Sep | 10 | Worc, LSC & LSF |
| | | | 12-18 Sep | 11 | Worc, LSC & LSF |

| | | |
|---|---|---|
| 19-25 Sep | 12 | Worc, LSC & LSF |
| 26 Sep-02 Oct | 13 | Worc, LSC & LSF |
| 03-09 Oct | 14 | Worc, LSC & LSF |

Worc-Worcester; LSC-Lakeside Container; LSF-Lakeside Circle

Table S2. Summary of the air samples sent for sequencing (metabarcoding)

| **Sample name** | **DNA conc (ng/µl)** | **DNA Amount (µl)** | **Sampling period** | **Location of sampling** |
|---|---|---|---|---|
| EE 2016 | 54.2 | 40 | 21 Jul-28 Sep 2016 | Worcester |
| LSC 2016 | 35.7 | 40 | 21 Jul-28 Sep 2016 | Lakeside Container |
| EE 2017 | 20 | 40 | 6 Jul-13 Sep 2017 | Worcester |
| LSC 2017 | 27.4 | 40 | 6 Jul-13 Sep 2017 | Lakeside Container |
| EE 2018 | 44.9 | 40 | 12 Jul-19 Sep 2018 | Worcester |
| LSC 2018 | 27.9 | 40 | 12 Jul-19 Sep 2018 | Lakeside Container |
| LSF 2018 | 21.8 | 40 | 12 Jul-19 Sep 2018 | Lakeside Circle |
| EE 2019 | 15.8 | 56 | 5 Jul-9 Oct 2019 | Worcester |
| LSC 2019 | 13.8 | 56 | 5 Jul-9 Oct 2019 | Lakeside Container |
| LSF 2019 | 40.7 | 56 | 5 Jul-9 Oct 2019 | Lakeside Circle |
| Negative control (DES buffer) | 0 | 50 | N/A | N/A |
| Positive control (Mock community) | 25 | 50 | N/A | N/A |

**Key**

| | |
|---|---|
| EE | Edward Elgar |
| LSC | Lakeside Container |
| LSF | Lakeside Field/Circle |
| N/A | Not applicable |

Table S3. Summary of the number of reads and ASVs per sample and ITS barcode

| No | Sample name | Location | Sampling Environment | ITS1 | ITS2 | ITS1 | ITS2 |
|---|---|---|---|---|---|---|---|
| | | | | No. of reads | | No. of ASVs | |
| 1 | EE 2016 | Worcester | Urban | 82,843 | 66,190 | 1,658 | 511 |
| 2 | LSC 2016 | Lakeside Container | Rural | 83,810 | 51,439 | 1,602 | 401 |
| 3 | EE 2017 | Worcester | Urban | 106,330 | 75,581 | 1,747 | 512 |
| 4 | LSC 2017 | Lakeside Container | Rural | 104,439 | 72,073 | 2,402 | 527 |
| 5 | EE 2018 | Worcester | Urban | 92,727 | 68,679 | 320 | 611 |
| 6 | LSC 2018 | Lakeside Container | Rural | 116,955 | 60,206 | 1,284 | 466 |
| 7 | LSF 2018 | Lakeside Circle | Natural | 99,533 | 57,290 | 931 | 290 |
| 8 | EE 2019 | Worcester | Urban | 88,689 | 42,587 | 738 | 629 |
| 9 | LSC 2019 | Lakeside Container | Rural | 56,100 | 76,342 | 335 | 940 |
| 10 | LSF 2019 | Lakeside Circle | Natural | 94,893 | 73,629 | 1,708 | 482 |
| **Total** | | | | **926,319** | **644,016** | **12,725** | **5,369** |

Table S4a. (i) BLAST search result of the taxa detected for each ASV in ITS1

| ASV | DOI of reference sequence | Taxon | Score | E-value | Percent similarity | Mismatch | ASV sequence alignment start | ASV sequence alignment end | Reference sequence alignment start | Reference sequence alignment end |
|---|---|---|---|---|---|---|---|---|---|---|
| ASV5 | MT548676 SH1505864.08FU | *Alternaria infectoria* | 505 | 1.56E-141 | 99.6 | 0 | 1 | 276 | 5 | 281 |
| ASV10 | MT561399 SH1505864.08FU | *Alternaria infectoria* | 481 | 2.46E-134 | 100.0 | 0 | 1 | 260 | 20 | 279 |
| ASV25 | MT561399 SH1505864.08FU | *Alternaria infectoria* | 503 | 5.58E-141 | 99.6 | 1 | 1 | 275 | 20 | 294 |
| ASV29 | MT561399 SH1505864.08FU | *Alternaria infectoria* | 503 | 5.55E-141 | 100.0 | 0 | 1 | 272 | 20 | 291 |
| ASV36 | MT883454 SH1505864.08FU | *Alternaria infectoria* | 481 | 2.46E-134 | 100.0 | 0 | 1 | 260 | 12 | 271 |
| ASV55 | MT561399 SH1505864.08FU | *Alternaria infectoria* | 507 | 4.33E-142 | 100.0 | 0 | 3 | 276 | 1 | 274 |
| ASV72 | MK461061 SH1505864.08FU | *Alternaria infectoria* | 481 | 2.46E-134 | 100.0 | 0 | 1 | 260 | 39 | 298 |
| ASV82 | MT561399 SH1505864.08FU | *Alternaria infectoria* | 503 | 5.58E-141 | 99.6 | 1 | 1 | 275 | 20 | 294 |
| ASV88 | FJ627005 SH1505864.08FU | *Alternaria* sp. | 499 | 7.13E-140 | 100.0 | 0 | 3 | 272 | 2 | 271 |
| ASV89 | MT561399 SH1505864.08FU | *Alternaria infectoria* | 505 | 1.55E-141 | 100.0 | 0 | 1 | 273 | 20 | 292 |
| ASV93 | MT883454 SH1505864.08FU | *Alternaria infectoria* | 503 | 5.58E-141 | 99.6 | 1 | 1 | 275 | 12 | 286 |
| ASV100 | MT883454 SH1505864.08FU | *Alternaria infectoria* | 503 | 5.55E-141 | 100.0 | 0 | 1 | 272 | 12 | 283 |
| ASV124 | MT548683 SH1505864.08FU | *Alternaria infectoria* | 505 | 1.56E-141 | 99.6 | 0 | 1 | 276 | 5 | 281 |
| ASV147 | MT548676 SH1505864.08FU | *Alternaria infectoria* | 507 | 4.35E-142 | 99.6 | 1 | 1 | 277 | 5 | 281 |
| ASV207 | MT883454 SH1505864.08FU | *Alternaria infectoria* | 503 | 5.58E-141 | 99.6 | 1 | 1 | 275 | 12 | 286 |
| ASV217 | MT561399 SH1505864.08FU | *Alternaria infectoria* | 503 | 5.58E-141 | 99.6 | 1 | 1 | 275 | 20 | 294 |
| ASV225 | MT883454 SH1505864.08FU | *Alternaria infectoria* | 505 | 1.55E-141 | 100.0 | 0 | 1 | 273 | 12 | 284 |
| ASV467 | MW049129 SH1526411.08FU | *Alternaria* sp. | 453 | 5.35E-126 | 99.6 | 1 | 1 | 248 | 26 | 273 |
| ASV474 | MN394894 SH1526416.08FU | *Alternaria brassicae* | 451 | 1.93E-125 | 99.6 | 1 | 1 | 247 | 38 | 284 |
| ASV596 | MH861640 SH1505864.08FU | *Alternaria abundans* | 481 | 2.46E-134 | 100.0 | 0 | 1 | 260 | 115 | 374 |
| ASV690 | KX515772 SH1526398.08FU | Fungi | 449 | 6.92E-125 | 99.6 | 1 | 1 | 246 | 37 | 282 |
| ASV774 | KU752189 SH1524228.08FU | *Tamaricicola* | 518 | 2.06E-145 | 99.6 | 1 | 1 | 283 | 17 | 299 |
| ASV803 | UDB0768558 SH1526398.08FU | Eukaryote | 453 | 5.35E-126 | 100.0 | 0 | 1 | 245 | 10 | 254 |

DOI-Digital Object Identifier

Table S4a. (ii) Species hypotheses (SH) of the taxa detected for ASVs above

| **SH code** | **Taxon** | **Threshold (%)** | **No. of sequences** |
|---|---|---|---|
| SH2127331.08FU | *Alternaria metachromatica* | 0 | 199 |
| SH2127280.08FU | *Alternaria infectoria* | 0 | 372 |
| SH1157990.08FU | *Alternaria planifunda* | 3 | 13344 |
| SH1388252.08FU | *Alternaria eichhorniae* | 2 | 12551 |
| SH1526411.08FU | *Alternaria carotiincultae* | *1.5* | 93 |
| SH2180541.08FU | *Alternaria sonchi* | 0 | 4 |

Table S4b. (i) BLAST search results of the taxa detected for ASVs in ITS2

| ASV | DOI of reference sequence | Taxa | Score | E-value | Percent similarity | Mismatch | ASV sequence alignment start | ASV sequence alignment end | Reference sequence alignment start | Reference sequence alignment end |
|---|---|---|---|---|---|---|---|---|---|---|
| ASV3 | Y17066 SH1505864.08FU | *Alternaria infectoria* | 592 | 1.37E-167 | 100.0 | 0 | 1 | 320 | 244 | 563 |
| ASV6 | MW047051 SH1505864.08FU | *Alternaria* sp. | 592 | 1.37E-167 | 100.0 | 0 | 1 | 320 | 251 | 570 |
| ASV56 | UDB0762983 SH1526398.08FU | Eukaryote | 592 | 1.37E-167 | 100.0 | 0 | 1 | 320 | 370 | 689 |
| ASV75 | MH861640 SH1505864.08FU | *Alternaria abundans* | 590 | 4.90E-167 | 100.0 | 0 | 1 | 319 | 392 | 710 |
| ASV105 | Y17071 SH1526398.08FU | *Alternaria linicola* | 590 | 4.90E-167 | 100.0 | 0 | 1 | 319 | 215 | 533 |
| ASV123 | MW049129 SH1526411.08FU | *Alternaria* sp. | 580 | 2.93E-164 | 100.0 | 0 | 1 | 314 | 274 | 587 |
| ASV125 | LC440627 SH1526416.08FU | *Alternaria brassicae* | 588 | 1.76E-166 | 100.0 | 0 | 1 | 318 | 407 | 724 |
| ASV214 | MW008895 SH1505864.08FU | *Alternaria* sp. | 590 | 4.90E-167 | 100.0 | 0 | 1 | 319 | 243 | 561 |
| ASV222 | MW007755 SH1505864.08FU | *Mucor amphibiorum* | 592 | 1.37E-167 | 100.0 | 0 | 1 | 320 | 274 | 593 |
| ASV226 | MW047051 SH1505864.08FU | *Alternaria* sp. | 586 | 6.36E-166 | 99.7 | 1 | 1 | 320 | 251 | 570 |
| ASV408 | KR013208 SH1505864.08FU | *Alternaria triticina* | 584 | 2.28E-165 | 99.7 | 1 | 1 | 319 | 261 | 579 |
| ASV582 | Y17070 SH1526403.08FU | *Alternaria solani* | 599 | 8.28E-170 | 100.0 | 0 | 1 | 324 | 221 | 544 |
| ASV586 | UDB0750544 SH2750838.08FU | Fungi | 425 | 1.47E-117 | 95.5 | 12 | 1 | 266 | 244 | 509 |
| ASV678 | MN495826 SH1505866.08FU | *Alternaria brassicicola* | 586 | 6.30E-166 | 100.0 | 0 | 1 | 317 | 260 | 576 |
| ASV813 | UDB0750544 SH2750838.08FU | Fungi | 431 | 3.15E-119 | 95.9 | 11 | 1 | 266 | 244 | 509 |
| ASV985 | UDB0750544 SH2750838.08FU | Fungi | 431 | 3.16E-119 | 95.9 | 10 | 1 | 267 | 244 | 509 |
| ASV1039 | MK019176 SH3591419.08FU | *Alternaria* sp. | 494 | 3.97E-138 | 96.3 | 11 | 1 | 300 | 54 | 353 |
| ASV1387 | MK019176 SH3591419.08FU | *Alternaria* sp. | 444 | 4.05E-123 | 93.3 | 20 | 1 | 300 | 54 | 353 |
| ASV1497 | HQ873332 SH1505885.08FU | Pleosporaceae | 593 | 3.81E-168 | 100.0 | 0 | 1 | 321 | 254 | 574 |
| ASV1513 | MH864320 SH1505866.08FU | *Alternaria alternariae* | 580 | 2.93E-164 | 99.7 | 1 | 1 | 317 | 421 | 737 |
| ASV1682 | Y17069 SH1526403.08FU | *Alternaria solani* | 601 | 2.31E-170 | 100.0 | 0 | 1 | 325 | 221 | 545 |
| ASV1770 | MW047051 SH1505864.08FU | *Alternaria* sp. | 486 | 6.64E-136 | 94.1 | 19 | 1 | 320 | 251 | 570 |

Table S4b. (ii) Species hypotheses (SH) of the taxa detected for ASVs above

| **SH code** | **Taxon** | **Threshold (%)** | **No. of sequences** |
|---|---|---|---|
| SH1858972.08FU | *Alternaria metachromatica* | 0.5 | 890 |
| SH2127280.08FU | *Alternaria infectoria* | 0 | 372 |
| SH2127296.08FU | *Alternaria armoraciae* | 0 | 14 |
| SH1890287.08FU | *Alternaria eichhorniae* | 0.5 | 9284 |
| SH2179670.08FU | *Alternaria alternata* | 0 | 8122 |
| SH1157990.08FU | *Alternaria planifunda* | 3 | 13344 |
| SH1526411.08FU | *Alternaria carotiincultae* | 1.5 | 93 |
| SH2180541.08FU | *Alternaria sonchi* | 0 | 4 |
| SH2179718.08FU | *Alternaria brassicae* | 0 | 40 |
| SH2127283.08FU | *Alternaria rosae* | 0 | 164 |
| SH2127282.08FU | *Alternaria oregonensis* | 0 | 184 |
| SH1526403.08FU | *Alternaria venezuelensis* | 1.5 | 689 |
| SH1688435.08FU | *Alternaria tropica* | 1 | 495 |
| SH1890300.08FU | *Alternaria multirostrata* | 0.5 | 372 |
| SH2179682.08FU | *Alternaria cichorii* | 0 | 285 |
| SH1370004.08FU | *Alternaria tumida* | 2 | 923 |
| SH1858975.08FU | *Alternaria photistica* | 0.5 | 144 |
| SH2127285.08FU | *Alternaria mimicula* | 0 | 74 |
| SH2127286.08FU | *Alternaria obovoidea* | 0 | 54 |
| SH1890300.08FU | *Alternaria multirostrata* | 0.5 | 372 |
| SH2179682.08FU | *Alternaria cichorii* | 0 | 285 |
| SH3661280.08FU | *Alternaria* sp. | 0 | 1 |

Table S5. Summary of the individual taxa in the mock community and outcome of the taxonomic assignment to the individuals using UNITE eukaryotes and Fungal databases

| **Mock sample** | **Kingdom** | **Phylum** | **Class** | **Order** | **Family** | **Genus** | Eukaryote ITS1 **Species** | Fungi ITS1 **Species** | Eukaryote ITS2 **Species** | Fungi ITS1 **Species** |
|---|---|---|---|---|---|---|---|---|---|---|
| *Alnus glutinosa* | Viridiplantae | Anthophyta | Eudicotyledonae | Fagales | Betulaceae | *Alnus* | *firma* | | *fauriei* | |
| *Alternaria alternata* | Fungi | Ascomycota | Dothideomycetes | Pleosporales | Pleosporaceae | *Alternaria* | *tenuissima* | *tenuissima* | *eichhorniae* | sp. |
| *Alternaria sp.* | Fungi | Ascomycota | Dothideomycetes | Pleosporales | Pleosporaceae | *Alternaria* | *tenuissima* | *tenuissima* | *eichhorniae, sp.* | sp. |
| *Artemisia vulgaris* | Viridiplantae | Anthophyta | Eudicotyledonae | Asterales | Asteraceae | *Artemesia* | *sp., argyi* | | *sp., sieversiana* | |
| *Betula pendula* | Viridiplantae | Anthophyta | Eudicotyledonae | Fagales | Betulaceae | *Betula* | *nigra* | | *nigra* | |
| *Cladosporium sp.* | Fungi | Ascomycota | Dothideomycetes | Capnodiales | Cladosporiaceae | *Cladosporium* | *delicatulum* | *delicatulum* | *tenuissimum, sp.* | *tenuissimum* |
| *Corylus avenella* | N/A | N/A | N/A | N/A | N/A | N/A | N/A | N/A | N/A | N/A |
| *Dactylis glomerata* | Viridiplantae | Anthophyta | Monocotyledonae | Poales | Poaceae | *Dactylis* | *glomerata* | | *glomerata* | |
| *Lolium perenne* | Viridiplantae | Anthophyta | Eudicotyledonae | Poales | Poaceae | *Lolium* | *temulentum* | | *temulentum* | |
| *Phleum pratense* | Viridiplantae | Anthophyta | Eudicotyledonae | Poales | Poaceae | *Phleum* | *pratense* | | N/A | |
| *Platanus x hispanica* | Viridiplantae | Anthophyta | Eudicotyledonae | Proteales | Platanaceae | *Platanus* | *gentryi, rzedowskii, sp.* | | N/A | |
| *Quercus ilex* | Viridiplantae | Anthophyta | Eudicotyledonae | Fagales | Fagaceae | *Quercus* | *sp.* | | *pyrenaica, canariensis, ilex* | |
| *Quercus robur* | Viridiplantae | Anthophyta | Eudicotyledonae | Fagales | Fagaceae | *Quercus* | *pubescens, pontica* | | *frainetto, sp., robur* | |
| *Urtica dioica* | Viridiplantae | Anthophyta | Eudicotyledonae | Rosales | Urticaceae | *Urtica* | *gracilis* | | *massaica* | |

N/A- Not detected

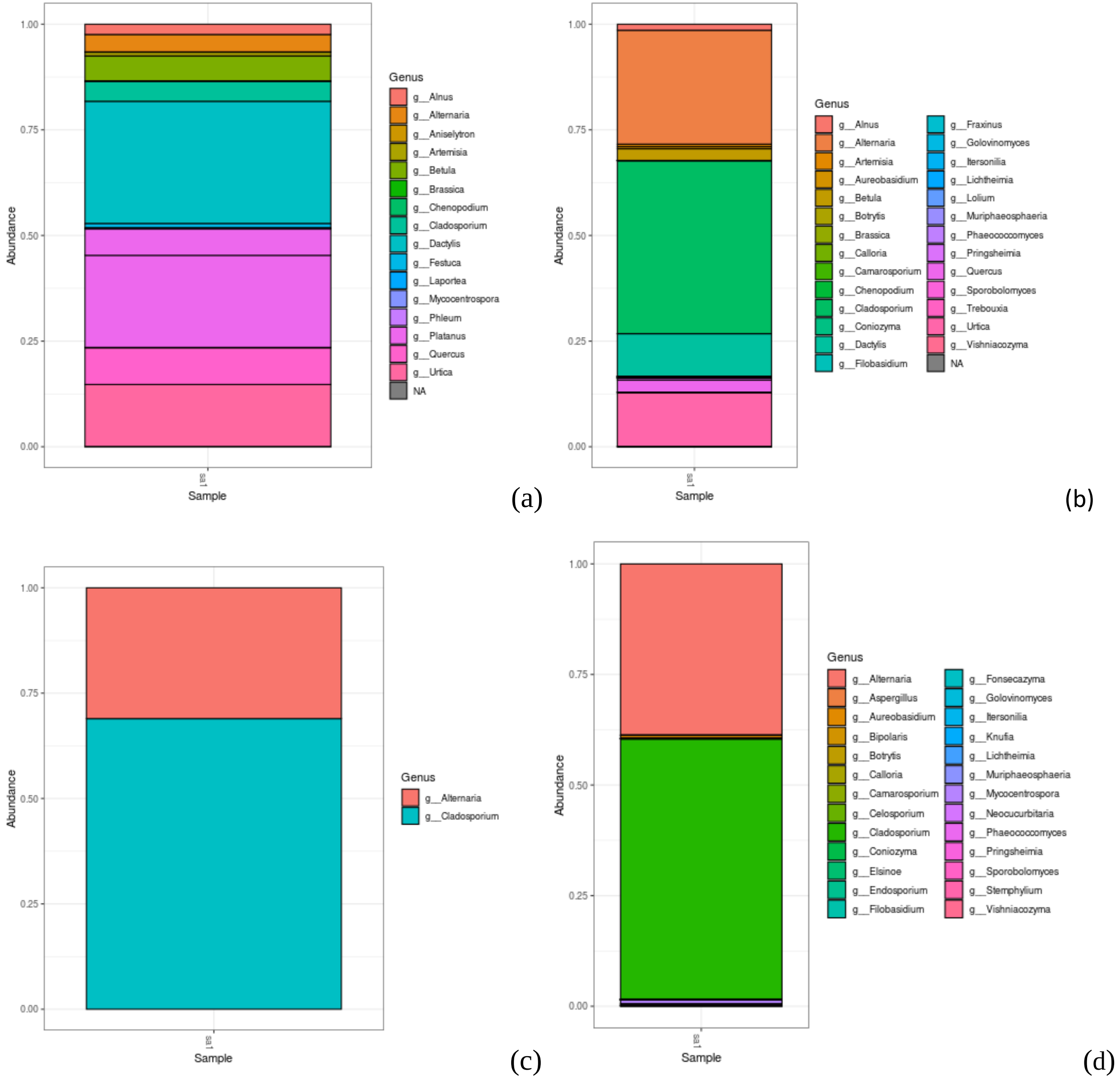


Fig. S1 Taxonomy, relative abundance (%) and diversity of the plant and fungal indididuals in the mock community at the genus level detected using (a & b) UNITE Eukaryotes database, ITS1 and ITS2 barcodes and (c & d) UNITE fungal database, ITS1 and ITS2 barcodes, respectively.

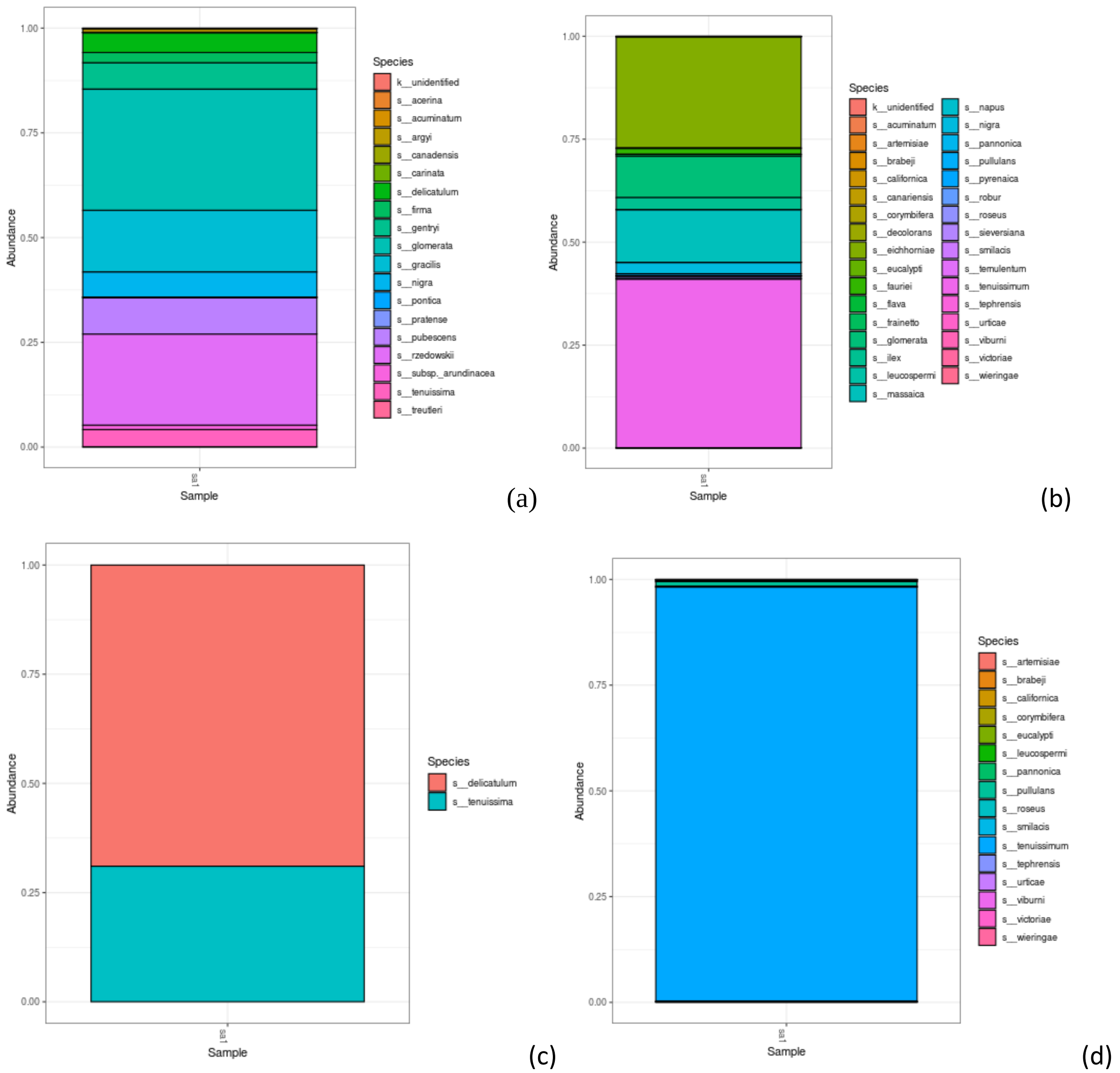


Fig. S2 Taxonomy and relative abundance (%) of the plant and fungal individuals in the mock community at the species level detected using (a & b) UNITE Eukaryotes database, ITS1 and ITS2 barcodes and (c & d) UNITE fungal database, ITS1 and ITS2 barcodes, respectively.
